# Network approach in liquidomics landscape

**DOI:** 10.1186/s13046-023-02743-9

**Published:** 2023-08-04

**Authors:** Daniele Santini, Andrea Botticelli, Antonio Galvano, Michele Iuliani, Lorena Incorvaia, Valerio Gristina, Chiara Taffon, Simone Foderaro, Elisa Paccagnella, Sonia Simonetti, Federico Fazio, Simone Scagnoli, Giulia Pomati, Francesco Pantano, Giuseppe Perrone, Elena De Falco, Antonio Russo, Gian Paolo Spinelli

**Affiliations:** 1 Oncologia Medica A, Policlinico Umberto 1, La Sapienza Università Di Roma, Rome, Italy; 2 Section of Medical Oncology, Department of Surgical, Oncological and Oral Sciences, University of Palermo, Palermo, Italy; 3 Medical Oncology, Fondazione Policlinico Universitario Campus Bio-Medico, Department of Medicine and Surgery, Università Campus Bio-Medico Di Roma, Selcetta, Italy; 4 Anatomical Pathology Operative Research Unit, Fondazione Policlinico Universitario Campus Bio-Medico, Rome, Italy; 5 Department of Medicine and Surgery, Research Unit of Anatomical Pathology, Università Campus Bio-Medico Di Roma, Rome, Italy; 6 Department of Medical Surgical Sciences and Biotechnologies, Sapienza University of Rome, C.So Della Repubblica 79, 04100 Latina, Italy; 7 UOC Oncologia Territoriale, Polo Pontino, La Sapienza Università Di Roma, Latina, Italy; 8 INI Casa Di Cura, Grottaferrata, Italy; 9 Mediterranea Cardiocentro, 80122 Naples, Italy

**Keywords:** Liquid biopsy, CTC, ctDNA, MRD, Targeted therapy, Liquidomics

## Abstract

Tissue-based biopsy is the present main tool to explore the molecular landscape of cancer, but it also has many limits to be frequently executed, being too invasive with the risk of side effects. These limits and the ability of cancer to constantly evolve its genomic profile, have recently led to the need of a less invasive and more accurate alternative, such as liquid biopsy. By searching Circulating Tumor Cells and residues of their nucleic acids or other tumor products in body fluids, especially in blood, but also in urine, stools and saliva, liquid biopsy is becoming the future of clinical oncology. Despite the current lack of a standardization for its workflows, that makes it hard to be reproduced, liquid biopsy has already obtained promising results for cancer screening, diagnosis, prognosis, and risk of recurrence.

Through a more accessible molecular profiling of tumors, it could become easier to identify biomarkers predictive of response to treatment, such as EGFR mutations in non-small cell lung cancer and KRAS mutations in colorectal cancer, or Microsatellite Instability and Mismatch Repair as predictive markers of pembrolizumab response.

By monitoring circulating tumor DNA in longitudinal repeated sampling of blood we could also predict Minimal Residual Disease and the risk of recurrence in already radically resected patients.

In this review we will discuss about the current knowledge of limitations and strengths of the different forms of liquid biopsies for its inclusion in normal cancer management, with a brief nod to their newest biomarkers and its future implications.

## Introduction

Behind the pathogenesis of cancer, there are accumulating mutations of genes involved in different pathways of cell survival, proliferation, and differentiation. Thus, currently, the way to identify their molecular profile, with important diagnostic and prognostic implications, usually consists of the direct tissue sampling of the tumor or metastatic lesion.

However, tumors are highly heterogeneous and sampling in their entirety is challenging, starting from the ability of their molecular profile to evolve over time. Several critical issues came out from the use of tissue sampling to determine the genomic profile of solid tumors such as the molecular divergency of individual cancers and metastatic lesions even within a single patient, and the molecular alterations induced by the therapeutic stress exerted by targeted drugs on tumor cells. Tissue biopsy is invasive, and it cannot be frequently repeated to monitor current tumor dynamics or response to treatment [1].

In contrast, the need for more sensitive and less invasive techniques to determine the molecular landscape of cancers has led to the development of genetic and genomic tests based on body fluids, especially from blood samples.

**Fig. 1:**
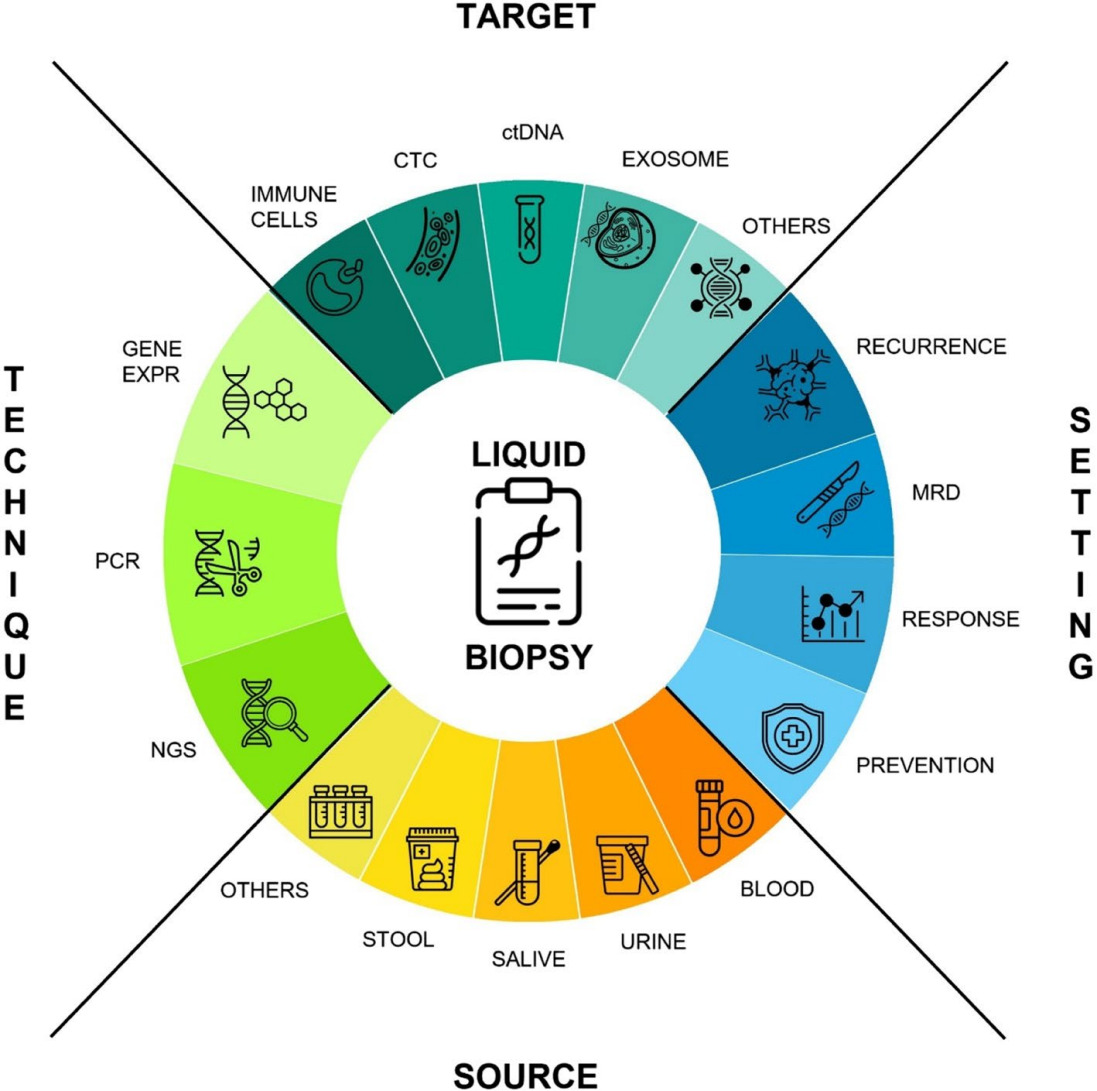
A schematized overview of the liquid biopsy with its targets, techniques involved, settings and sources of the samples

Liquid biopsies present different advantages over standard diagnostic tissue biopsy (Fig. 1): they are minimally invasive, having a simpler and more convenient sample and fewer side effects for patients, and potentially leading to more accurate prediction of tumor incidence, progression, treatment response, and survival prognosis [2–4].

The primary marker analyzed through liquid biopsies are distinctive tumor-derived components: circulating tumor cells (CTCs), cancer cells that leave the primary tumor potentially invading other tissues through the bloodstream [5, 6]; cell-free DNA (cfDNA), that has already presented raised levels in the serum of cancer patients and was first described by Mandel and Metais in 1948 [7, 8]; circulating tumor DNA (ctDNA), a fraction of cfDNA that belongs to cancer and presents its mutations [9, 10], studied for its implications as a prognostic and predictive factor for patients and for cancer detection [11–13]; tumor-derived RNAs (i.e. mRNA and miRNA) [14, 15]; extracellular vesicle, such as exosomes, of recent interest [16].

Moreover, blood is not the only body fluid that can be analyzed by liquid biopsy, extending the sources of cancer-derived molecules to other fluids such as urine [17], saliva [18], and stools [19].

The development of a targeted approach to investigating ctDNA, which studies known genetic mutations located in specific genes, has led to important progress for targeted therapies, such as the ability to predict therapeutic response to the EGFR inhibition in lung cancer by analyzing specific mutations of this gene [11, 20, 21]. On the other hand, an untargeted approach, aiming to detect any unknown mutation through whole genome sequencing, can lead to the discovery of new biomarkers involved in cancer management and prognosis. Detection of ctDNA can also be relevant for the identification of minimal residual disease (MRD) even in the absence of clinical evidence in patients following curative treatment or surgery [22, 23].

Anyways, liquid biopsy still presents some issues that must be considered to improve the evidence of its clinical utility, especially due to the lack of standardization across workflows during the different phases of laboratory testing, from specimen collection to its analysis.

Herein, we provide a brief overview of the various advantages and the current limitations of liquid biopsy in the management of cancer. We will also discuss the old and newest biomarkers and techniques implicated in its utility in cancer diagnosis, prognosis, and monitoring of treatment response or recurrence, including several promising studies that recently came out to enlighten how liquid biopsy should be integrated even more in clinical practice.

## Technical aspects: limits and perspectives (sampling, storage, technologies, PCR, NGS, CGP, etc.), structured reports

Liquid biopsy for cancer patients involves the isolation of circulating tumor cells, circulating tumor DNA, and other tumor-derived materials such as proteins and exosomes from patient blood samples. Circulating tumor DNA (ctDNA) represents promising biomarkers in cancer diseases. ctDNA can be isolated from many body fluids, such as blood, saliva, urine, ascites, bile, cerebrospinal fluids, and pleural effusion may be considered as a source of ctDNA [1].

Despite the advantages of liquid biopsy, the majority of assays still lack evidence of clinical utility and validity [24], with only four tests [25] obtaining approval from the Food and Drug Administration (FDA). One reason for this is that liquid biopsy assays often lack reproducibility [26] due to the absence of standardization across workflows. For clinical labs to successfully implement liquid biopsy, they need to develop easy-to-use, robust, and reproducible workflows [27] that include “standard operating procedures” across all phases of laboratory testing. Of particular interest is the standardization of pre-analytical workflows for liquid biopsy as assay outcome can be influenced by many different variables during this phase.

The pre-analytical phase of liquid biopsy (Table 1) includes all the steps prior to analysis such as specimen collection, stabilization, transport, enrichment, processing, and isolation and quality assessment of the analyte. The purpose of this workflow is to maintain the integrity of the sample following blood draw and prepare it for analysis [28]. The pre-analytical phase is arguably the most important part of liquid biopsy workflows as 46% to 68% of errors occur during this phase [29]. These errors can adversely affect data quality in the following phases and can result in incorrect treatment decisions [29].

**Table 1:** Preanalitic variable in liquid biopsy

Variables	Pitfalls	Recommendation
Patient condition	- concentrations of cfDNA increase between physio-pathological conditions: autoimmune diseases, trauma, strenuous exercise, pregnancy - in most early-stage cancers, the amount of cfDNA is very low, similar to healthy subjects	- LB sensibility is higher in patients with high tumor burden
Type of cancer	different tumor types do not release the same amount of ctDNA	- LB sensibility is higher in patients with metastatic cancers of the pancreas, bladder, colon, stomach, breast, liver, esophagus, head and neck and melanoma
Type of blood collection tubes used	risk of WBCs lysis, leading again to ctDNA contamination with wild-type background DNA	- K2/K3EDTA-containing tubes require a short time interval (< 6 h) between blood drawing and sample processing - Specialized blood collection tubes containing a preservative agent maintain stable cfDNA levels for 7 days if stored at RT
Blood processing protocol used	Reduction of cfDNA yield	Double centrifugation step: the first at 1,600 xg, the second at 16,000 xg, 10 min each at 4° C
Plasma storage	Long periods of plasma storage may cause a decreased cfDNA yield	plasma storage for 2 weeks at -20 °C or 4 weeks at -80 °C has no effect on cfDNA extraction
ctDNA storage	Long periods of ctDNA storage may cause DNA fragmentation	Storage ctDNA extracts at -20 °C or preferably at -80 °C, avoid more than three freeze–thaw cyckes
Quality assessment method	Potential false positives are due to clonal hematopoiesis	Assays should incorporate sequencing of leukocytes in addition to plasma DNA

Arechederra M et al. reviewed the literature comparing different methodological approaches for each step in the sample preparation process [28]. The sheer number of reports combined with the sometimes-contradictory impacts of different pre-analytical variables highlights the urgent need to standardize these procedures [24]. To standardize these aspects of the pre-analytical phase, researchers first need to understand their impact on sample integrity and the eventual success of liquid biopsy tests [30].

Blood withdrawal represents one of the best sources due to the very simple and minimally invasive way of sampling. Moreover, it can be repeated at different time points, giving the opportunity for real-time monitoring of the disease. Circulating Free DNA (cfDNA) are spread from both cancer and normal cells, but in cancer patients their concentrations are greater [31, 32]. Circulating tumor DNA (ctDNA) is part of the cfDNA deriving from the tumor mass.

In cancer patients, a proportion of these cfDNA molecules also derive from the primary and secondary tumors. Although it was originally thought that the higher level of cfDNA in the blood of cancer patients might be a cancer biomarker itself, it has been since shown that many other conditions result in similar cfDNA increase. In this regard, important points must be considered: i) concentrations of cfDNA vary enormously between individuals and their physio-pathological conditions, being increased not only in advanced cancer patients but also in other scenarios including, autoimmune diseases, trauma, strenuous exercise, or pregnancy; ii) in most early stage cancers, the amount of cfDNA is very low, similar to healthy subjects [33]; iii) the fraction of ctDNA fragments in the total cfDNA is very small, varying from less than 0.01% to over 10% according to tumor burden [34] and tumor metabolism [35]; iv) different tumor types do not release the same amount of ctDNA, and, even in patients with the same disease, the concentration of ctDNA may vary consistently. In fact, Bettegowda et al. showed that most disease patients with metastatic cancers of the pancreas, bladder, colon, stomach, breast, liver, esophagus, and head and neck, as well as patients with neuroblastoma and melanoma, harbored detectable levels of ctDNA. In contrast, less than 50% of patients with metastatic cancers of the kidney, prostate, or thyroid harbored detectable ctDNA [36].

Many different pre-analytical aspects can lead to interlaboratory variability when performing liquid biopsy. These variables include i) the type of blood collection tubes used, ii) the storage conditions of the blood sample, iii) the time between blood collection and sample processing, iv) the blood processing protocol used, v) the extraction method used, vi) and the quality assessment method used. The impact of each of these variables depends on the liquid biopsy application [37–39].

Since blood is the most used source for ctDNA, plasma represent the matrix preferred in the majority of clinical trials and EDTA containing tubes are used for blood collection [37, 38, 40]. Using these tubes clotting is inhibited, and thus it is possible to recover plasma that represent the matrix of choice for ctDNA extraction. Actually, also serum can be used as a matrix to isolate ctDNA; indeed, it has been reported that the amount of ctDNA in serum can be 2–24 times higher than in plasma. This can be a consequence of the clotting process that causes white blood cells (WBCs) breaking, finally leading to the release of wild-type DNA. This contamination causes a further dilution of the tumor-specific DNA, making it even more difficult to detect.

Another important pre-analytical aspect is the time that elapses between the withdrawal and its processing for plasma recovery. Indeed, the more time passes, the more is the risk of WBCs lysis, leading again to ctDNA contamination with wild-type background DNA.

To prevent this increase in genomic DNA, blood samples stored in EDTA tubes that will be analyzed for circulating tumor DNA need to be processed within 6 h after the blood draw [41]. To overcome the inconvenience caused by this time restriction, there is a growing list of stabilizing reagents and dedicated blood collection tubes designed to preserve cell-free DNA profiles in whole blood [42]. These tubes prevent cell lysis, limiting contamination of the sample with genomic DNA. Blood samples for circulating tumor DNA analysis stored in specialized tubes can be kept at room temperature for a number of days before processing is needed [43].

While researchers have made progress in understanding how the type of tube used and storage conditions impact circulating tumor DNA analysis, no consensus on best practices has yet been reached [28]. There are also many other pre-analytical variables whose impacts on ctDNA analysis are unknown. Information on how these variables impact other applications for liquid biopsy, such as exosome analysis, remains unclear [24].

Another aspect to be considered is the high turnover ctDNA (15 min half-life), therefore some authors suggested to proceed with plasma preparation by centrifugation within 1 h after blood collection [40, 44].

Concerning sample processing, the complete removal of any cellular component is essential. For this goal, the best option is a two-step centrifugation at 1600 g for 10 min for plasma isolation [45]. According to this recommendation, Herrera et al. reported less concentration of cfDNA in plasma samples that were centrifuged twice compared with samples that were centrifuged only once (13 µg/l vs. 819 µg/l), revealing that cfDNA concentrations were contaminated with genomic DNA [46]. These observations confirm that the second centrifugation step is crucial for ctDNA analysis. Finally, it is well known that ctDNA integrity is better conserved as cfDNA extracts compared to plasma when samples are stored at -80 °C and avoiding freeze–thaw cycles [38].

As regard methods for ctDNA isolation, Sorber L et al. [47] have compared the efficiency of the most used kit, the QIAamp circulating nucleic acid kit (QIA), with four other cfDNA isolation kits: the PME free-circulating DNA Extraction Kit (PME), the Maxwell RSC ccfDNA Plasma Kit (RSC), the EpiQuick Circulating Cell-Free DNA Isolation Kit (EQ), and two consecutive versions of the NEXTprep-Mag cfDNA Isolation Kit (NpMV1/2). In the study, the detection of KRAS mutation and total cell-free DNA concentration were performed with droplet digital PCR, whereas real-time PCR was used to evaluate cfDNA integrity. They showed that QIA and the RSC kits displayed similar isolation efficiencies, whereas the yield generated by the PME and NpMV2 kits was significantly lower [47].ctDNA investigation can be achieved through two different analytical approaches: a targeted approach and an untargeted approach. The targeted approach relies on the possibility to analyze known genetic mutations that occur in hotspot region of specific genes with implications for therapy decisions. Among these methods, we can include real-time PCR, droplet digital PCR (ddPCR) and targeted next-generation sequencing (NGS).

In the untargeted approach, it is possible to investigate ctDNA without the knowledge of any specific mutations present in the primary tumor. This can be achieved through whole genome sequencing using NGS platforms. Nevertheless, this analysis is quite expensive and sometimes difficult to interpret; thus, it can be used for biomarkers discovery in the context of disease monitoring, detection of molecular resistance, and identification of new therapeutic targets. Despite whole genome sequencing, a more cost-effective method in the exome sequencing, which does not require prior knowledge of the genetic landscape of the tumor.

The main targeted approaches are real-time PCR, ddPCR and targeted NGS [48]. Real-time PCR represents the oldest technique and the power of this technique in detecting mutant allele at a very low frequency (< 1%) is limited, and therefore other more sophisticated methods have been developed. In ddPCR, the partitioning is obtained through an emulsion PCR, each generated droplets ideally represent a PCR reactor. At the end of the analysis, software allows to identify a positive or a negative signal indicating the presence or absence of a target sequence. Therefore, mutated ctDNA can be detected in a wide background of wild-type sequences. The ddPCR platforms now available are various, each of them with a more or less different workflow, but they all share a very high sensitivity (0.01%) [49].

NGS has revolutionized our approach to molecular testing, indeed we can analyze multiple genes and multiple patients at a time with a consistent reduction in time and money. Of great interest, there is the paper of Newman et al. that has developed cancer personalized profiling by deep sequencing (CAPP-Seq) (10.1038/nm.3519). CAPP-Seq method is able to detect ctDNA in 100% of patients with stage II–IV non–small-cell lung carcinoma and in 50% of patients with stage I. The diagnostic specificity was 96% for mutant allele fractions down to approximately 0.02% [50].

Several international organizations are working toward developing standards for liquid biopsy workflows. These organizations are either working directly to build these standards or are developing the infrastructure needed for data sharing across stakeholders to reach a consensus.

SPIDIA4P (https://www.spidia.eu/) is a continuation of SPIDIA, which tackled the standardization and improvement of pre-analytical procedures for in vitro diagnostics. The next phase of the initiative involves working to improve the global health care system by developing selected high-priority pre-analytical European Committee for Standardization (CEN) and International Organization for Standardization (ISO) standard documents. They are also looking to develop corresponding External Quality Assessment (EQA) schemes and implementation tools.

CANCER-ID (https://www.cancer-id.eu/) is a European consortium that is working to establish standard protocols for blood-based biomarkers. They are also working to clinically validate such biomarkers. This consortium is funded by the Innovative Medicines Initiative and is composed of 36 partners from 13 countries.

BloodPAC (https://www.bloodpac.org/) is an American initiative to accelerate the development, validation, and clinical use of liquid biopsy assays in order to better inform medical decisions so that patient outcomes can be improved. They have developed a collaborative infrastructure that allows for information sharing between stakeholders in the public, industry, academia, and regulatory agencies. They hope that information sharing, and evidence generation will help bring liquid biopsy into routine clinical practice.

An important step in the delivery of precision oncology to patients with lung cancer is the interpretation and reporting of variants in the clinical context [51]. Certain minimum requirements are needed for the reporting of molecular profiling results for all CAP-accredited laboratories [52]. These requirements cover assay methodology, basic clinical performance characteristics including clinical and analytical sensitivity and specificity, assay results, and interpretation. Recently, the ESMO Precision Medicine Working Group published recommendations (Table 2) on the use of circulating tumour DNA for patients with cancer [53].

**Table 2:** Recommendation for a structured report

Clinical Data	- cancer diagnosis - disease stage - treatment at time of acquisition
Timing	- data (dd/mm) and time (hh/mm) of blood sample - data (dd/mm) and time (hh/mm) of plasma separation
Tubes used	- K2/K3EDTA-containing tubes - specialized blood collection tubes containing preservative agent
Result	- variants detected related to the clinical request - VAF for each variants detected - if a variant is not detected should be reported as “non-informative” or “not detected” rather than “negative”
Potential germline variants	Potential pathogenic germline variants in genes associated with heritable cancer predisposition should be flagged with an alert for the clinician
Variants potentially associated with CHIP	Variant identified in ctDNA assay is assumed to be present in the tumour but could be derived from leukocytes
Variant allele fractions for quantitative assays	Variant type and/or genomic features detected by assay SNVs, small insertions/deletions, amplifications, copy number losses, gene fusions, MSI, TMB and LOH
Technology used for analysis	- Q-PCR - dd-PCR - Mass Spettrometry - NSG
Kits used for the analysis	IVD or IVD-R certificated kits should be used
Limit of detection	In cases where input plasma DNA is limiting, a warning should be inserted in the report
Assay limitations	ctDNA results have an amount of discordance with tumour testing. The report should communicate this potential discordance

All LB reports should contain date of sample acquisition, type of tubes used, timing of plasma separation, method and timing of ctDNA extraction. Moreover, treatment exposure (on/off treatment) at time of acquisition should be reflected.

Cases where gene variants are not detected must be reported as ‘non-informative’ or ‘not detected’, instead of ‘negative’. Indeed, ctDNA assays have an appreciable rate of discordance with tumour testing. Cases where a mutation is not detected may be interpreted as the variant not being present in the tumour, when in actuality, there was insufficient ctDNA in the specimen. Report communicates the potential for discordance in such cases.

Variant allele fractions (VAF) may provide information suggestive of possible germline origin, clonal relatedness of variants in the same panel and the potential for a false-positive result. ctDNA samples with low VAF variants can be the most challenging aspect of reliably reporting ctDNA results [54, 55]. Indeed, with the use of highly sensitive NGS approaches (LOD ∼0.5% or lower), somatic mutations within nonmalignant hematopoietic cells, known as clonal hematopoiesis, might represent a source of “biological noise” in cell-free DNA analyses.

Moreover, in patients with low disease burden or with bone or brain metastasis, circulating free DNA (cfDNA) quantities may be low. Moreover, some specific mutations can be under-representative of their frequency in tumors such as KRAS G12 [56]. It is unknown whether variants at low allele fractions are as responsive to targeted therapy as those at high allele fractions. Some studies indicated that low VAF oncogenic drivers respond to targeted therapy, which serves to emphasize the need for highly sensitive tests [57].

Variants in genes commonly implicated in clonal hematopoiesis of indeterminate potential (CHIP) should be flagged to caution the clinician about the potential non-tumour origin of these variants [58]. Clonal haematopoiesis is a common challenge for assays that include genes implicated in clonal haematopoiesis. Variant identified in ctDNA assay is assumed to be present in the tumour but is actually derived from leukocytes. Report should communicate the potential non-tumour origin of variants in genes commonly implicated in CHIP.

Targeted variant or regions examined by assay should be reported. This could range from a single variant for digital PCR assays (e.g. EGFR, c.2369C > T, p.T790M) to hundreds of genes for an expanded NGS-based panel. Assays are validated to detect and report specific types of variants (e.g., SNVs, small insertions/deletions, amplifications/copy number losses, gene fusions). Report should communicate which variant types are reported.

The limit of detection for each variant type should be determined and reported, ideally with an associated confidence interval. Some variant types are more difficult to detect with ctDNA assays. Report should communicate individual performance of different variant types. In cases where input plasma DNA is limiting, the reported sensitivity is adjusted, or a warning is inserted in the report.

Specific tumor variants identified should be classified as ‘actionable’ or “not”. Benign lesions can contain oncogenic variants. Identification of an oncogenic variant in ctDNA assays is not diagnostic of malignancy. As an example, BRAF V600E variant has been identified in plasma DNA from individuals with benign nevi [59]. Interpretation of ctDNA assays should be done in the context of tissue studies and other clinical information. To support classification, the Association for Molecular Pathology (AMP), American Society of Clinical Oncology (ASCO), and College of American Pathologists (CAP) jointly published a four-tiered system classification system for the interpretation and reporting of sequence variants in cancer [60]. The European Society for Medical Oncology (ESMO) also recommends the ESMO Scale for Clinical Actionability of Molecular Targets (ESCAT) variant classification guidelines, with subtle differences from the AMP/ASCO/CAP Guidelines [61].

## Role of liquid biopsy in heredo-familiar tumors

The essential component of cancer risk assessment is the preventive oncology trough screening and early diagnosis [62]. About 5–10% of cancers have a hereditary component where specific and heritable pathogenic variants are clearly implicated in the genesis of the disease. Over 300 hereditary cancer susceptibility syndromes are reported [63], involving both families and individuals tested for mutation carriers [64].

Cancer predisposition-related genes may be classified into 3 groups based on penetrance: high (lifetime cancer risk: 50% or greater), moderate (lifetime cancer risk: 20% to 50% or a 2–fourfold increase above the general population risk), and low or unknown risk.

Currently, testing options for the identification of germinal mutation include single-gene testing and/or cancer panels. There are also two major categories of NGS cancer panels: cancer-site-specific panel testing and pan-cancer panel testing [63]. There are some screening methods proved to be useful for cancer prevention in high-risk phenotypes [65], as for breast, ovarian, pancreatic and colorectal cancer. However, limitations are based on low sensitivity and specificity and normally applicable to a single cancer type [62]. Despite the consolidated and progressive introduction of the genomic profiling in our daily practice in oncology by NGS and the advent of personalized oncology [63], minimally invasive approaches for the early diagnosis and the monitoring and prediction of the therapeutic response in cancer patients [66], are under intensive investigation, also in light of the intra and inter-tumor heterogeneity accompanied by dynamic biological changes and the sub-clonal genome architecture occurring over the time, which represent the most significant diagnostic challenge in the cancer field with unavoidable implication in clinic.

As a suggestion of possible germline origin, in a series of 1000 consecutive patients who underwent tissue NGS, 2.3% of patients were discovered to be carriers of a previously unrecognized germline mutation [67]. Although somatic and germline variants should be readily distinguished based on VAF, in a small subset of patients with high ctDNA burden this may not be possible and patients should be informed of the possibility that high-risk germline variants may be incidentally detected in a liquid biopsy. The informed consent should clarify whether the patient wants to be informed about these incidental findings. Reporting of potential germline variants should generally follow ESMO recommendations for germline-focused analysis of tumour-only sequencing [68]. Patients identified with a previously unrecognized germline mutation should be promptly referred for genetic counselling [52].

Specific features of hereditary cancer syndromes are related to higher frequency of classical genetic disorders, early clinically onset, and very likely potential risks to develop additional neoplasms.

Besides, a pool of genes with a certain degree of penetrance rather than a single genomic alteration, often influences the evolution of the disease. In this context, the investigation and the diagnostic validation of liquid biopsy likely finds its best application, as patients with inherited syndromes undoubtedly implies a narrower clinical surveillance [69].

For instance, the Lynch syndrome (LS, also known as hereditary non-polyposis colorectal cancer syndrome, HNPCC), which is inherited in an autosomal dominant pattern and accounting of the 3–5% of colorectal cancers, is caused by genomic mutations of the mismatch repair system (MMR), whose detection is a key step to screen this set of patients and possibly to combine the immunotherapy regimen.

Coherence of MMR phenotype between tumor tissue and cell free DNA (cfDNA) obtained through liquid biopsy, has been reported in subjects with LS [66]. To date, cfDNA obtained from liquid biopsies is suitable for detecting MMR mutations, microsatellite instability (MSI) and MLH1 promoter methylation status, and universal CRC markers.

There are also other biomarkers proposed for the LS screening, as blood sampling is not the only form of liquid biopsy providing ctDNA. Mutations in the telomerase reverse transcriptase (*TERT*) promoter and the fibroblast growth factor receptor 3 (*FGFR3*) are often found in LS. These alterations have been proposed as novel biomarkers of urothelial cancer (UC), the third most common cancer type in certain subsets of LS families and they are ideal candidates to be studied from ctDNA extracted from urine liquid biopsy. Bile is another source of ctDNA, as almost 4% of LS patients develop bile duct cancer [66].

Similarly, cell free DNA, found in patients with pancreatic cancer, has been demonstrated to possess a diagnostic/predictive significance: cfDNA is present at diagnosis in almost 50% of these patients with localized disease and that circulating tumor DNA may anticipate of 6.5 months potential recurrences [70]. This aspect is significant as almost 20% of prostate cancer cases show a familial origin history [71]. Other reports have shown that the detection rate of circulating DNA in pancreatic cancer, depends on the technique employed. When genomic alterations of a specific gene is sought (i.e. KRAS), a clear discrepancy between tissue and liquid biopsy is found [72], therefore suggesting that liquid biopsy requires the suitable technique in order to strengthen its diagnostic potential.

However, not only free DNA is currently investigated for inherited syndromes. Coherently, the novel concept of “circulome”, which entails miRNAs, mRNA, RNA, exosomes, extracellular vesicles (EV) and metabolites, has becoming a novel diagnostic strategy [73, 74]. The circulome can be considered the novel frontier of the liquid biopsy. The detection based more on a defined pool of molecules of cancer origin rather than relying on a single biomarker, is useful to design a more precise molecular scenario exhibited by the patient. For instance, the combination of the pathogenic variants of BRCA1/2 and high levels of two circulating proteins SPARC (Secreted protein acidic and rich in cysteine) and THBS1 (Thrombospondin 1), can be combined to distinguish women with ovarian cancer from those healthy and with wild type BRCA1/2 variants [75].

Thus, genomic and protein alterations are better integrated, allowing to reveal new insights on the heterogeneous facets of cancer. Bioinformatic algorithms and array analysis have been recently applied to the circulome, simplifying the predictive significance in hereditary cancers and overcoming the limitations of the small amount of soluble molecules and biomarkers often difficult to detect [72].

Circulating mRNA and miRNAs related to MMR can also be employed for the same purpose with an enhanced sensitivity and useful to stratify patients [66], therefore discriminating between patients with sporadic alterations of the MMR from those with LS. Notably, researchers are exploring differentially expressed miRNAs, which are more stable in the body fluids [76–78], but also their methylation status for follow ups or correlation to chemoresistance, therefore expanding the field of applicability in genetic-associated cancer disorders.

The epigenetic change such as methylation of circulating free tumor DNA, miRNAs or proteins is considered a key mechanism involved in the early tumorigenesis, therefore a useful screening and predictive tool [79]. The Circulating Cell-Free Genome Atlas Study (CCGA) based on the deep sequencing of methylation of circulating cell-free nucleic acids (cfNAs) is currently under attention for its potential to discriminate cancer *vs* non cancer (NCT02889978) [80].

Accordingly, the combined methylation analysis of both A disintegrin and metallopeptidase with ADAMTS1 (thrombospondin type 1 motif 1) reflects high sensitivity for cancer pancreatic diagnosis, increasing even more at higher stages of the tumor [81].

Moreover, EV have been studied in pancreatic cancer at early stages, by investigating the cargo of miRNAs, proteins and specific molecules such as the proteoglycan GPC1 (Glypican-1) found in serum of patients and revealed as a marker with high sensitivity of detection [82]. Despite this, we are still far from using EV as diagnostic/prognostic platform, given a wide range of biological variability among studies and technique employed [72].

Additional biological sources might implement the early detection of pancreatic cancer as demonstrated for driver genomic mutations of KRAS (G12V and G12D) found in pancreatic juice before malignancy is proven [83]. Notably, combining the detection of multiple genomic mutations with the size of mutated DNA fragments in the liquid biopsy and the stage of cancer, has been found useful to discriminate patients from healthy subjects.

However, several techniques are attempting to ameliorate the amplification, the mutational analysis or the methylation status of the small amount of free DNA in the blood. These are not limited to NGS-based systems but may include digital droplet PCR, and the inter-Alu-PCR or even nano-magnetic platforms [84] to enhance the sensitivity and reduce false negative samples. In addition, the detection of the mitochondrial DNA mutations in liquid biopsy seems to be a promising biomarker for the diagnosis of early colorectal cancer risk [85].

Sequencing-based technology combined with liquid biopsy (specifically with cell free DNA) such as the PapGene test, has been currently set up for screening of subjects with inherited predisposition to gynaecological cancers, LS and germline mutations in BRCA1, 2 or MMR system [86, 87], demonstrating that the diagnostic significance of the liquid biopsy can be strengthen by associating high throughput molecular platforms. Some clinical trials regarding liquid biopsy-based approaches in LS and breast cancer (detection of BRCA1 both in blood or circulating tumor cells of women with mutated TP53 mutation detection), are already completed (NCT02198092 and NCT02608346, respectively).

Other example of non-yet FDA approved combination of liquid biopsy with NGS is the Guardant360 (Guardant Health) and FoundationOne Liquid (Foundation Medicine), considered as companion diagnostic tests employed for prostate, breast, and ovarian cancers. There is evidence that the matching of NGS and liquid biopsy could help to improve the stratification of patients, attempting to understand who can really benefit from the targeted therapy expecially in advanced cancers, as demonstrated in metastatic breast cancer [88].

Liquid biopsy can also provide indications regarding potential actionable targets identified within multiple gene-based panels besides the canonical genomic mutations. For instance, alterations in ERS1 (Estrogen Receptor 1) gene, which is associated to oestrogen resistance, has been found in circulating tumor DNA of a cohort of patients with breast cancer [89]. Women with advanced hormone-receptor-positive and HER2 negative breast cancer eligible for therapy with alpelisib (active in patients with PIK3CA mutations), exhibit in the circulome (specifically in cDNA, EV and circulating tumor cells) PIK3CA mutations, mirroring the genomic alterations found in the corresponding cancer tissue [90].

A key question is how liquid biopsy can change the landscape of the therapy.

## Role of liquid biopsy in minimal residual disease

Despite initial success of radical treatment of early-stage tumors, a substantial number of patients develops virtually incurable distant metastases during a variable period of time. Minimal Residual Disease, namely the presence of disseminated cells in the organism without clinical or radiological signs of disease, determines this fait accompli [91]. Neoadjuvant and adjuvant treatments have shown to improve long-term outcomes and are thus the standard of care for many tumors. However, those therapies are administered to every patient statistically considered to be at reasonable risk for distant recurrence in absence of tangible prove of cancer dissemination, thus most treated patients are exposed to toxicities without any benefit. The assessment of MRD by random sampling of organs trough tissue biopsy for all patients would obviously be unfeasible.

In this scenario, liquid biopsy is nowadays the most promising tool being implemented to unveil MRD, trough detection of shed circulating tumor products, like cells (CTCs) [92], DNA (ctDNA) [93] or RNA (ctRNA) [94]. Baseline and longitudinal repeated sampling of blood from radically resected patients could enable the detection of impending disease ahead of clinical and radiological methods and could be used to better define the real risk of relapse, helping the clinicians decide whether to start a treatment. Furthermore, the molecular characterization of circulating tumor material could be used to better define appropriate treatment. The relapse, especially for breast cancer, can happen years later from the dissection of primary tumor. However, tumors are made of cells bearing distinct molecular signatures. This inevitable heterogeneity is the result of the forces that initiate and promote normal cell transformation and represents the key feature that determines treatments failure [95]. Despite solid biopsy being feasible most of the time, they are invasive procedures and hardly repeatable in everyday clinical setting. Being a non-invasive and easily repeated tool, liquid biopsy is destined to help us keep pace with tumor evolution.

Nowadays the use of liquid biopsy to assess MRD has yet to enter in clinical practice (Table 3), but many studies have proven its ability to better define the prognosis of radically operated patients in a large number of solid tumors.

**Table 3:** Potential liquid biopsy applications in MRD setting

Prognostic value	Basal and after-surgery liquid biopsy assessment could be used as a marker of higher risk disease and increased events of disease recurrence or death, to guide the choice of (neo)adjuvant treatment administration or omission
Recurrence monitoring	Liquid biopsy has proven to be more sensitive in detecting early disease recurrence compared to standard methods during follow-up. Its use could be implemented in everyday clinical practice to treat relapses as soon as they present, even in absence of overt metastases
Liquid biopsy as a measure of early liquid recurrence	During adjuvant treatments, monitoring liquid biopsy elements levels could help determine early recurrence and consequently influence the choice of new therapeutic strategies
Patients’ treatment selection based on molecular alterations: the predictive value of liquid biopsy	Liquid biopsy could be used to select a population harboring genetic or epigenetic alterations that could be targetable by a biological therapy

### Prognostic and systemic treatment need definition

One of the major challenges in oncology is defining the population of radically resected patients that cannot be cured by surgery alone and that needs the administration of systemic therapy to eradicate the chances of relapse. A large and growing body of literature (Tables 4 and 5), has highlighted the grim prognostic value of MRD identified by liquid biopsy in patients that underwent surgery, pointing out a clearly positive correlation between the presence of residual tumor cells and the risk of relapse and death. Furthermore, clinical trials have initiated considering liquid biopsy as a tool to decide whether to start an adjuvant treatment, introducing a possible paradigm shift in everyday clinical practice.

**Table 4:** Key studies of prognostic CTCs analyses

Study (ref.)	Tumor type	Timing of blood withdrawal	Number of patients	Detection method	Prognostic relevance
Bidard et al. 2010 [96]	BC	before and after neoadjuvant chemotherapy	115	Immunocytochemical (CellSearch system)	Pretreatment CTC detection was an independent, strong prognostic factor for OS in nonmetastatic breast cancers during neoadjuvant chemotherapy and even a single CTC detected in 7.5 ml of blood was associated with the subsequent development of metastases
Rack et al. 2014 [97]	BC	Before and after adjuvant chemotherapy	2026	Immunocytochemical (CellSearch system)	Independent prognostic relevance of CTCs both before and after adjuvant chemotherapy
Janni et al. 2016 [98]	BC	After adjuvant treatments (chemotherapy ± OT) or neoadjuvant treatment (chemotherapy)	3173	Immunocytochemical (CellSearch system)	The presence of CTCs was an independent predictor of poor disease-free, overall, breast cancer–specific, and distant disease-free survival
Riethdorf et al. 2017 [99]	BC	Before and after neoadjuvant chemotherapy	213	Immunocytochemical (CellSearch system)	Detection of CTCs in blood collected before NAT was associated with reduced DFS and OS whereas CTCs detected after NAT were not
Bidard et al. 2018 [100]	BC	After neoadjuvant chemotherapy	1574	Immunocytochemical (CellSearch system)	Number of CTCs detected before NAT had a detrimental and decremental effect on OS, DDFS and LRFS
Sparano et al. 2018 [101]	BC	After adjuvant treatments (chemotherapy ± OT)	547	Immunocytochemical (CellSearch system)	CTC positivity was associated with a 13.1-fold higher risk of recurrence; 4.1% of patients with hormone receptor-negative disease had CTCs detected, none of whom had disease recurrence
Goodman et al. 2018 [102]	BC	After adjuvant treatments (chemotherapy ± OT followed by radiotherapy)	3213	Immunocytochemical (CellSearch system)	CTC status was predictive of a benefit of RT for LRFS, DFS, and OS in patients treated with surgery followed by systemic therapy
Trapp et al. 2018 [103]	BC	Before and after adjuvant chemotherapy	1087	Immunocytochemical (CellSearch system)	CTC status 2 years after chemotherapy was independently prognostic of OS and DFS
Bidard et al. 2021 ([104]bi)	BC	Before adjuvant treatment (OT ± chemotherapy)	778	Immunocytochemical (CellSearch system)	CTC count may be a reliable biomarker method for guiding the choice between chemotherapy and endocrine therapy as the first-line treatment in hormone receptor–positive, ERBB2-negative metastatic breast cancer
Matikas et al. 2022 [105]	BC	Before and after adjuvant chemotherapy	1220	Real-Time PCR	CTC positivity at baseline was associated with shorter DSF and OS
Van Dalum et al. 2015 [106]	CRC	Before surgery	183	Immunocytochemical (CellSearch system)	the presence of CTC is associated with a statistically significant higher risk of disease recurrence and shorter RFS and a higher colon cancer related death. Presence of CTC also has a significant impact on the disease course when measured 2 to 4 years after surgery but not within the first year after surgery
Hinz et al. 2017 [107]	CRC	Before surgery	299	Real-Time PCR	Detection of CTC in the blood was correlated with a significantly worse 5-year OS and DFS rate
Dizdar et al. 2019 [108]	CRC	After surgery	31	GILUPI CellCollector (CC) and CellSearch system (CS)	No significant correlation with clinicopathological parameters or overall survival was observed with CC CTCs. In contrast, detection of CTCs with CS was significantly correlated with reduced overall survival
Krebs et al. 2011 [109]	NSCLC	Before and after chemotherapy	101	Immunocytochemical (CellSearch system)	Among stage III-IV patients, those with ≥ 5 CTCs after one cycle of chemotherapy had a significantly worse prognosis than those with fewer than 5 CTCs. Furthermore, CTC number was modulated by therapeutic intervention in 18 patients who presented positive for CTCs at baseline, and changes in CTC numbers after therapy seemed to be correlated with PFS (P < 0.001) and OS (P = 0.009
Hou et al. 2012 [110]	SCLC	Before chemotherapy for limited stage SCLC	31	Immunocytochemical (CellSearch system)	failure of CTC number to decrease to less than 50 after one cycle of chemotherapy is associated with worse prognosis
Dorsey et al. 2015 [111]	NSCLC	Before, during and after radiotherapy	30	telomerase-based assay	CTC counts appeared to reflect the clinical course and response to treatment
Chinniah et al. 2019 [112]	NSCLC	Before and after chemoradiation	48	telomerase-based assay	detectable CTC levels in many patients meaningfully precede radiologic evidence of disease recurrence
Frick et al. 2020 [113]	NSCLC	Before and after radiotherapy	92	telomerase-based assay	high pre-SBRT CTC count and persistence of CTCs were both associated with regional/distant recurrence
Kuske et al. 2016 [114]	PCa	before and three months after radical prostatectomy	86	CellSearch system, in vivo cellCollector, EPISPOT	CTC detection by EPISPOT before radical prostectomy significantly correlated with PSA serum values and clinical tumor stage, while the other assays showed no significant correlations
Salami et al. 2019 [115]	PCa	Before radical treatment (prostectomy or radiotherapy)	45	Epic Sciences platform	recurrence and metastasis were associated with significant differences in baseline CTC detection. Patients experiencing biochemical recurrence had significantly greater numbers of AR-positive CTCs, and patients developing metastases had significantly more total CTCs and AR-positive CTCs
Rink et al. 2012 [116]	UCB	Before surgery	100	Immunocytochemical (CellSearch system)	CTC status was an independent predictor of disease recurrence, cancer-specific mortality and all-cause cause mortality
Gazzaniga et al. 2012 [117]	NIMBC	Before surgery	44	Immunocytochemical (CellSearch system)	Presence of CTC was found significantly associated to shorter time to first recurrence
Gazzaniga et al. 2014 [118] Nicolazzo et al. 2019 [119]	NIMBC	Before surgery	102	Immunocytochemical (CellSearch system)	CTC presence predicted both decreased time to first recurrence and time to progression. An updated analysis revealed that CTC predicted shorter CSS and OS
Busetto et al. 2017 [120]	NIMBC	Before surgery	155	CellSearch system and CELLection Dynabeads	there was a strong correlation between CTC presence and time to first recurrence. Time to progression was also strongly correlated with CTCs
Abrahamsson, J. et al. 2017 [121]	UCB	before surgery in patients treated solely with cystectomy. In patients given preoperative chemotherapy, a sample was collected before commencement of chemotherapy, and an additional sample was taken before cystectomy in patients who were CTC positive before chemotherapy	75	CellSearch system	presence of CTCs was associated with an increased risk of progression among patients treated with radical cistectomy with or without perioperative chemotherapy. However, an increased risk of cancer-specific death was not observed for patients with CTCs
Soave et al. 2017 [122]	UCB	Before surgery	226	CellSearch system	patients with presence of CTC had reduced recurrence-free, cancer-specific, and overall survival, compared to patients with absence of CTC
Beije et al. 2022 [123]	UCB	Before surgery	273	CellSearch system	OS did not statistically significantly differ between CTC-negative and CTC-positive patients. The cancer-specific survival in CTC-positive patients was significantly shorter than that in CTC-negative patients. Disease relapses occurred significantly more in CTC-positive patients than in CTC-negative patients

**Table 5:** Key studies of prognostic ctDNA analyses

Study (ref.)	Tumor type	Timing of blood withdrawal	Number of patients	Detection method	Prognostic relevance
Olsson et al. 2015 [124]	BC	After surgery during follow-up	20	WGS of primary tumors and quantification of tumor-specific rearrangements in plasma by ddPCR	post-surgical ctDNA monitoring enabled accurate discrimination between patients with and those without distant recurrence
Garcia-Murillas et al. 2015 [23]	BC	Before neoadjuvant therapy, after surgery and then every 6 months during follow-up	55	personalized dPCR assay	the detection of ctDNA was correlated with an increased risk of metastatic relapse
Chen et al. 2017 [125]	BC	After surgery and during adjuvant therapy	38	Oncomine Research Panel	33 patients had at least one mutation identified in their primary tumour, only 4 of whom had mutations detected in cfDNA. the 4 patients with detectable ctDNA had disease relapse within 9 months
Riva et al. 2017 [126]	BC	before neoadjuvant therapy; after 1 cycle; before surgery; after surgery	46	customized ddPCR probes	slow decrease of ctDNA level during NCT was strongly associated with shorter survival
McDonald et al. 2019 [127]	BC	Before, during and after neoadjuvant therapy	33	targeted digital sequencing (TARDIS)	ctDNA concentrations were lower in patients who achieved pathological complete response (pathCR) compared to patients with residual disease
Coombes et al. 2019 [128]	BC	Every 6 months for 4 years after surgery	49	personalized assays targeting 16 variants selected from primary tumor whole-exome data	plasma ctDNA was detected ahead of clinical or radiologic relapse in 16 of the 18 relapsed patients; metastatic relapse was predicted with a lead time of up to 2 years
Garcia-Murillas et al. 2019 [129]	BC	Before neoadjuvant therapy; after surgery	101	personalized dPCR assay	detection of ctDNA at diagnosis and during follow-up was associated with worse relapse-free survival. Brain-only metastasis was less commonly detected by ctDNA
Parsons et all 2020 [130]	BC	After surgery	142	WES was used to identify patient-specific single-nucleotide variants. Patient-specific SNVs were used to design custom MRD tests, which were subsequently applied to cfDNA and germline DNA libraries	MRD detection at 1 year was strongly associated with distant recurrence
Magbanua et al. 2021 [131]	BC	Before and during neoadjuvant therapy and before surgery	84	personalized ctDNA test to detect up to 16 patient-specific mutations	Lack of ctDNA clearance was a significant predictor of poor response and metastatic recurrence
Marla Lipsyc-Sharf, 2022 [132]	BC	After surgery	103	WES on primary tumor tissue was used to identify somatic mutations tracked via a personalized ctDNA test (RaDar)	ctDNA was identified a median of 1 year before all cases of distant metastasis
Tie et al. 2016 [22]	CRC	After surgery and after adjuvant therapy (two different cohorts)	230	NGS-based assay	ctDNA detection identified patients at very high risk of recurrence
Ng et al. 2017 [133]	CRC	Before and after surgery. After recurrence	44	patient-specific ctDNA assays based on multiplexed detection of somatic mutations identified from patient primary tumours	ctDNA was detected in 11 of 15 patients at or before the time of clinical or radiological recurrence of CRC
Schøler et al. 2017 [134]	CRC	Before and after surgery	27	Personalized ddPCR assays based on WES of primary tumor	patients treated with curative intend for localized disease who were ctDNA-positive within the first postoperative trimester had a very high risk (100%) of relapsing
Reinert et al. 2019 [135]	CRC	Before and after surgery	130	NGS-based assay	During surveillance after definitive therapy, ctDNA-positive patients were more than 40 times more likely to experience disease recurrence than ctDNA-negative patients
Tarazona et al. 2019 [136]	CRC	at baseline, 6–8 weeks after surgery, and every 4 months for up to 5 years	150	Personalized ddPCR assays based on WES of primary tumor	Detection of ctDNA after surgery and in serial plasma samples during follow-up were associated with poorer disease-free survival. In patients treated with adjuvant chemotherapy, presence of ctDNA after therapy was associated with early relapse
Taieb et al. 2019 [137]	CRC	After surgery	805	ctDNA was tested by using the detection of 2 methylated markers (WIF1 and NPY) by ddPCR	A notable improvement in the disease-free survival of patients who had detectable ctDNA postoperatively and received a longer duration (6 months vs 3 months) of adjuvant chemotherapy was demonstrated
Tie et al. 2021 [138]	CRC	After surgery	485	SafeSeqS	ctDNA detection was associated with poorer 5-year recurrence-free and overall survival
Parikh et al. 2021 [139]	CRC	After surgery or adjuvant therapy	103	Guardant Reveal test	In plasma drawn 1-month after definitive therapy and > 1 year follow-up, 15 patients had detectable ctDNA, and all 15 recurred.Of 49 patients without detectable ctDNA at the landmark timepoint, 12 recurred
Vidal et al. 2021 [140]	CRC	Before neoadjuvant therapy and before surgery	72	Guardant Reveal test	Detectable presurgery ctDNA was significantly associated with systemic recurrence, shorter disease-free survival and shorter overall survival
Henriksen et al. 2022 [141]	CRC	Before and after surgery and after adjuvant therapy	168	NGS-based assay	Detection of ctDNA was a strong recurrence predictor postoperatively and directly after ACT. The recurrence rate of postoperative ctDNA-positive patients treated with ACT was 80%. Only patients who cleared ctDNA permanently during ACT did not relapse. Serial ctDNA assessment after the end of treatment was similarly predictive of recurrence. The ctDNA growth rate was prognostic of survival
Tie et al. 2022 [142]	CRC	After surgery	455	SafeSeqS	A ctDNA-guided approach to the treatment of stage II colon cancer reduced ad-juvant chemotherapy use without compromising recurrence-free survival
Abbosh et al. 2017 [143]	NSCLC	After surgery	24	NGS-based, patient-specific mutational panel assays	the detection of SNVs in ctDNA seemed to be correlated ith clinical evidence of NSCLC relapse
Chaudhuri er al. 2017 [144]	NSCLC	Before and after surgery and during follow-up	40	CAPP-Seq	ctDNA was detected in the first post-treatment blood sample, within 4 months of primary treatment, in 94% of patients with subsequent recurrence
Chen et al. 2019 [145]	NSCLC	Before surgery, after tumor resection, after surgery and during follow-up		cSMART	The ctDNA detection on the third day after R0 is associated with higher risk of relapse and mortality
Xia et al. 2022 [146]	NSCLC	Before and after surgery	330	NGS-based	Preoperative ctDNA positivity was associated with lower recurrence-free survival. The presence of MRD (ctDNA positivity at postoperative 3 days and/or 1 month) was a strong predictor for disease relapse. MRD-positive patients who received adjuvant therapies had improved RFS over those not receiving adjuvant therapy, whereas MRD-negative patients receiving adjuvant therapies had lower RFS than their counterparts without adjuvant therapy
Gale et al. 2022 [147]	NSCLC	Before and after surgery and during follow-up	88	WES on primary tumor tissue was used to identify somatic mutations tracked via a personalized ctDNA test (RaDar)	Detection within 2 weeks to 4 months after treatment end occurred in 17% of patients, and was associated with shorter recurrence-free survival and overall survival. ctDNA was detected 1–3 days after surgery in 25% of patients yet was not associated with disease recurrence. Detection before treatment was associated with shorter overall survival and recurrence-free survival
Lau et al. 2020 [148]	PCa	Before and after surgery	8	Personalized ddPCR assays based on WGS of primary tumor	ctDNA was identified in 2 of 8 patients. Both of them had primary PSA persistence and very rapid disease trajectories, characterised by early progression to overt metastatic disease and death
Powles et al. 2021 [149]	UCB	At the start of adjuvant therapy	581	Personalized ddPCR assays based on WES of primary tumor	ctDNA testing at the start of therapy identified patients who had poor prognosis. Notably, patients who were positive for ctDNA had improved disease-free survival and overall survival in the atezolizumab arm versus the observation arm

Tie et al. assessed the role of ctDNA in defining stage II CRC prognosis and real need for adjuvant therapy [142]. Patients were randomly assigned to have treatment decisions guided by either ctDNA results or standard clinicopathological features. The results showed how ctDNA-guided decision for adjuvant treatment led to lower therapy administration (15% vs. 28% in the control group) without statistically significant differences in the 2-year RFS (93.5% and 92.4% in the control group).

Powles et al. evaluated ctDNA levels in patients enrolled in the IMvigor010 trial, that randomized patients to receive atezolizumab or observation after surgical resection for operable urothelial cancer [149]. The study did not show significant advantage in the active arm neither in DSF nor in OS [150]. However, when stratifying the patients based on the presence of ctDNA, improved disease-free survival and overall survival in the atezolizumab arm versus the observation arm was observed for ctDNA patients positive. For ctDNA negative patients, there was again no meaningful difference between arms.

These pioneering trials show that a liquid-biopsy-enhanced stratification of patients is possible and is likely to better select patients for active versus observational approaches. An increasing number of trials is ongoing to further develop this fundamental clinical question (NCT05411809; NCT04259944; NCT03748680; NCT04089631).

It is therefore possible that, in the future, adjuvant therapy will be escalated for ctDNA positive patients and standard or not administered at all for ctDNA negative patients. To further define the need for escalation of treatments in ctDNA positive patients, in the IDEA trial the presence of postoperative ctDNA was tested as a prognostic and predictive marker for prolonged adjuvant treatment duration [137]. ctDNA was confirmed as an independent prognostic marker and treatment for 6 months was superior to 3 months in both ctDNA negative and ctDNA positive patients. ctDNA positive patients treated 6 months had a similar prognosis to ctDNA negative patients treated 3 months. Trials with escalated treatment in ctDNA positive versus standard treatment in ctDNA negative resected patients are ongoing (NCT05062889; NCT04803539; NCT05427669).

### Recurrence monitoring

Follow-up of radically resected patients is an integrated part of clinical oncology routine but evidence regarding the effectiveness of the different follow-up strategies varies substantially. The identification of relapse as soon as it presents, even in the absence of overt metastases, could maximize the changes of cure or at least delay complications related to the tumoral mass presence. Blood withdrawal is a guideline-included procedure for many tumors, especially those for which an oncological marker is recognized, thus the introduction of liquid biopsy would not pose a problem for patients. Despite few information is available regarding the prognostic relevance of liquid biopsy analyses focused on the surveillance of MRD through follow-up care studies, findings indicate that the detection of CTCs and ctDNA can provide evidence of metastatic relapse earlier than standard procedures.

To address this clinical question, Reinert et al. longitudinally analyzed ctDNA in a cohort of 125 stage I, II and III colon cancer [135]. Data showed that ctDNA-positive patients at postoperative day 30 had a higher recurrence rate compared with those who were ctDNA negative after surgery. Similarly, ctDNA positivity in patients treated with adjuvant chemotherapy was associated with a high risk of recurrence. Moreover, serial ctDNA analysis during surveillance after definitive treatment identified relapse with 88% sensitivity and 98% specificity. Interesting, ctDNA analyses revealed disease relapse up to 16.5 months ahead of standard-of-care computed tomography. These results clearly suggest that clinical applications of ctDNA in CRC could improve risk stratification, adjuvant chemotherapy monitoring and early relapse detection.

Similarly, Tarazona et al. performed a longitudinal evaluation of plasma ctDNA in 94 early CRC patients before and after the surgery [136]. Data showed that ctDNA presence, after surgery and during follow-up, were correlated with worse disease-free survival. In addition, ctDNA detection in patients after adjuvant chemotherapy was associated with early relapse. Detection of ctDNA had a median of 11.5-months lead time over radiological relapse suggesting the utility of ctDNA in identifying MRD and patients at high risk of disease recurrence.

The IMPROVE-IT2 (NCT04084249) is an ongoing trial that compare post-operative surveillance by ctDNA analysis or standard-of-care CT-scan in radically resected CRC patients [151]. The hypothesis is that combining ctDNA analysis and radiological assessments could improve the early detection of recurrent disease optimizing the postoperative treatment.

### Liquid biopsy as a measure of response

Response to adjuvant therapy is impossible to assess with normal clinical and radiological exams, being the aim of the treatment to cure invisible MRD. Therefore, adjuvant treatment is administered, when possible, at its higher intensity, without the possibility to monitor the real effectiveness of the ongoing therapy. For patients that will eventually relapse, this means being exposed to toxicities that are sometimes fatal without any benefit. Furthermore, adjuvant regimens are always interrupted after a defined number of cycles, without real clue of the disease state at that point. All these limitations could be surpassed by MRD monitoring through liquid biopsy during and after treatment. We have already shown how monitoring ctDNA after adjuvant treatment can identify patients that convert to a negative status and are therefore at less risk of relapse from those that remain positive and have thus a worse prognosis.

Key findings come also from Henriksen et al., that investigated post-adjuvant chemotherapy ctDNA status in stage III colon cancer patients [141]. In particular, ctDNA presence was associated with disease recurrence postoperatively also in patients treated with adjuvant chemotherapy. Only patients who showed permanent clearance of ctDNA after adjuvant therapy did not relapse. Serial ctDNA analysis after the end of treatment was also predictive of disease recurrence suggesting that ctDNA assessment has a strong prognostic value.

For those patients in which ctDNA levels do not lower during and/or after treatment, if clinically feasible, one of those 3 options should be considered, given the proven grim association within ctDNA presence and relapse: switch of the treatment to another regimen, its prolongation or intensification, when possible, with addition of biomarker-based therapy in those patients with an actionable alteration.

The concept of a “second line adjuvant treatment” represents an absolute paradigm shift from today’s clinical practice. This approach, aimed to cure and not to palliate, presents obvious advantage for the patients, as the toxicities from therapies could be better tolerated without the burden of the metastatic disease. Furthermore, tumors are less resistant to therapies when the cells are isolated and scattered. Two trials (NCT04567420; NCT04985266) are currently investigating a second line adjuvant treatment for high-risk resected breast cancer patients currently undergoing hormonal treatment. Primary objective of the therapeutic randomized phase is to assess whether palbociclib plus fulvestrant improves relapse-free survival compared to standard of care adjuvant endocrine therapy in patients with detectable ctDNA in the plasma but without evidence of metastatic disease on imaging. Another trial (NCT05343013) is defining if TAS-102 treatment in resected colon cancer patients with positive ctDNA after completion of adjuvant chemotherapy treatment can determine a 6-month ctDNA clearance. In NCT04920032 trial, the percent of patients positive for ctDNA after 6 cycles or at least 3 months after starting second line adjuvant treatment will be used to estimate the efficacy of adjuvant trifluridine and TAS-102 in combination with irinotecan in patients with ctDNA positive colon adenocarcinoma after first line standard adjuvant treatment. The NCT05062889 trial aims to evaluate two different aspects in colon cancer resected patients: the escalation treatment for ctDNA positive patients (FOLFOXIRI vs FOLFOX/CAPOX in ctDNA negative) and the ctDNA clearance induced by TAS-102 in ctDNA positive patients after first line adjuvant therapy.

### Patients’ treatment selection based on molecular alterations

Liquid biopsy-guided treatment based on molecular alterations is already consolidated clinical practice, especially for breast and lung cancers, in the metastatic settings [152, 153]. Several tests are already utilized and approved [153]. Guardant360 CDx test was FDA approved as a companion diagnostic for patients with *EGFR*-mutant NSCLC, with *EGFR* exon 20 insertion NSCLC and with *KRAS* G12C mutations NSCLC who may benefit from treatment with Osimertinib, Amivantamab and Sotorasib, respectively. Foundation Medicine’s FoundationOne Liquid CDx is approved as a companion diagnostic for the poly (ADP ribose) polymerase inhibitor rucaparib for the treatment of advanced metastatic prostate cancer and ovarian cancer with *BRCA1/2* mutations, as a companion diagnostic to identify patients with *BRCA1/2* mutations and/or *ATM* alterations in metastatic colorectal cancer for whom treatment with olaparib may be appropriate, to identify *ALK* rearrangements in patients with NSCLC eligible for treatment with alectinib as well as three tyrosine kinase inhibitors, including gefitinib, osimertinib, and erlotinib, approved for the first-line treatment of *EGFR*-mutant NSCLC, to assess TMB and MSI status in NSCLC and to identify mutations in the *PIK3CA* gene in patients with breast cancer eligible for treatment with alpelisib.

However, the introduction of blood molecular testing in the early setting is still in development and only few small trials are currently investing its role. One of such trials (NCT05079022) aims to assess the role of Furmonertinib, a third generation anti-EGFR, in EGFR-mutated radically resected stage I lung cancers, with the mutation being detected trough ctDNA analysis. The primary end point is the clearance of ctDNA at 6 months. Another study (NCT05388149) plans to escalate therapy in Her2-positive, radically resected with residual invasive disease following prior neoadjuvant trastuzumab (± pertuzumab)-based chemotherapy, breast cancer patients with the addition of Neratinib to TDM-1, if ctDNA is detected in plasma. The primary endpoint is again the clearance of ctDNA. As shown, clearance of ctDNA demonstrated to increase survival in radically resected patients after adjuvant treatment, but it’s validity as a surrogate endpoint for overall survival has still to be proven.

As tissue-based analysis for detection of molecular disease have already entered the clinical practice, for example for guiding anti-EGFR adjuvant treatment in NSCLC or anti-BRCA adjuvant treatment in breast cancer, the possibility of tracking the emergence of resistance mutations to a given treatment by liquid biopsy is becoming more and more appealing.

## Role of Liquid biopsy in agnostic indications

Recently, some drugs have been approved regardless of the primary tumour type, but solely on the basis of fundamental molecular abnormalities driving the processes of carcinogenesis and disease progression. This innovative approach of precision medicine led to the first agnostic approvals of oncology drugs [154] (Tables 6 and 7).

**Table 6:** Applications of liquid biopsy in Agnostic therapy

Target	Methods	Findings	Challenges	References
bTMB	Foundation Medicine bTMB assay	High bTMB was associated with greater ORR and a trend toward increasing OS and PFS benefit in patients with NSCLC treated with first-line atezolizumab	-lack of standardisation in the technique for detecting bTMB -lack of standardization in defining cut-off points for high bTMB - lack of evidence in several type of cancers	[155, 156]
MSI/dMMR	Guardant360® CDx and the liquid CDx FoundationOne	A high degree of concordance between tissue-based MSI determination and MSI determination based on circulating tumour DNA has been reported in the literature	- Detection limits due to low disease burden, location of metastasis or concurrent treatment (chemotherapy/radiotherapy) - most evidence of accuracy found in colorectal cancer	[157, 158]
NTRK re-arrangements	Plasma based NGS-assay	-In a retrospective study the NTRK1 fusion detected by ctDNA was confirmed in tissue in 88% of cases - plasma-based NGS tests demonstrated high concordance with tissue genotyping in several reports including NTRK genes fusion in the panel	Lack of previous reports in literature evaluating the role of cfDNA analysis in NTRK fusion positive solid tumours	[159, 160]
BRAF mutation V600E	- NGS platform - Idylla platform, real-time PCR based test	- High sensitivity and specifity - concordance between plasma and tissue analysis	Most of the literature concerns colon- rectal cancers, NSCLC and melanoma	[161–166]

*bTMB* blood Tumor mutational burden, *ORR* Overall response rate, *PFS* Progression free survival, *OS* Overall survival, *dMMR* deficiency of DNA mismatch repair, *MSI* Microsatellite instability, *NGS* Next generation sequencing, *NTRK* Neurotrophic receptor tyrosine kinase, *NSCLC* No small cell lung cancer

**Table 7:** Agnostic therapy: take home messages

Molecular Markers	Take home messages
TMB	● The determination of TMB on peripheral blood is not yet standardised in the absence of a well-defined cut-off ● Further studies are needed to confirm the reliability of liquid biopsy in determining TMB compared to tissue analysis
MSI	● Actually two NGS-based approches are FDA-approved blood-based diagnostic tests and are considered suitable for the determination of MSI on peripheral blood samples
NTRK fusion	● Currently the potential of liquid biopsy in identifying NTRK fusions should be further explored ● In some reports, plasma-based NGS tests have shown a high degree of concordance with tissue genomic tests for several genetic mutations, including NTRK fusions
BRAF	● Most of the published literature on the clinical use of liquid biopsy to detect patients with BRAF mutation concerns maily mCRC, melanoma and NSCLC, while few data are available on less frequent types of cancer ● Liquid biopsy in the determination of Braf mutations should be further explored in patients with different types of solid tumours
PI3K mutation	● Further studies are needed to assess whether alpelisib may have an agnostic indication in solid tumours carrying the PI3KCA mutations ● Liquid biopsy has been extensively studied and currently approved to detect PI3CA-mutated breast tumours

Further trials to validate and standardise analysis techniques in solid tumours are urgently needed to expand the use of liquid biopsy in clinical practice for the agnostic indications

In the last years, scientific research has focused on identifying biomarkers predictive of response to immunotherapy. The deficiency of DNA mismatch repair (dMMR) and MSI were among the first biomarkers used as expressing tumour mutability. Based on the results of five independent clinical trials (Keynote-016, Keynote-164, Keynote-012, Keynote-028, and Keynote-158), pembrolizumab received its first FDA approval for the treatment of adult and paediatric patients with unresectable or metastatic solid tumours, MSI-High (MSI-H) or dMMR, progressing after standard treatments and lacking other treatment options [167, 168].

Furthermore, in 2020 the FDA expanded the approval of pembrolizumab to include unresectable or metastatic tumors with high tumor mutational burden that have progressed following prior treatment and that have no satisfactory alternative therapy options. The FDA also approved the FoundationOneCDx assay as a companion diagnostic test for pembrolizumab [169].

The neurotrophic receptor tyrosine kinase (NTRK) genes, including NTRK1, NTRK2 and NTRK3, are key regulators of neuronal and embryonic development. NTRK rearrangements were shown to be able to drive oncogenesis, independently of histology [170, 171]. Indeed, NTRK fusions were detected in several type of solid tumors, such us, lung, breast, pancreatic, colon and thyroid [172]. On the basis of a combined analysis of three clinical trials, NCT02122913, NCT02637687 and NCT02576431, which included cancer patients with fusion in one of the three known NTRK genes, larotrectinib was the first FDA-approved molecule in November 2018 for adult and paediatric patients with NTRK fusions solid tumours [173]. The second TRK and ROS1 inhibitor molecule was Entrectinib, approved in August 2019, as an additional therapeutic option for NTRK fusion-positive tumours [174, 175].

BRAF is a gene encoding for a member of the Raf family, which plays a central role in many cell proliferation and differentiation processes through the MAP kinase (MAPK) pathway [176].

Mutated BRAF gene may be a key oncogenic driver in promoting carcinogenesis and tumour progression [177].

The Cancer Genome Atlas (TCGA) has identified BRAF mutations in many tumour types, especially melanomas, thyroid cancers, lung cancers. However, this mutation could also occurs in rare histological tumour types [178], such as diffuse gliomas, cholangiocarcinoma, hairy cell leukaemia, multiple myeloma and Langerhans cell histiocytosis [179].

In August 2022, the FDA approved the combination of dabrafenib (Tafinlar) and trametinib (Mekinist) for adult and paediatric patients (6 years of age or older) with unresectable or metastatic BRAF V600E-mutant solid tumours that have progressed after previous treatment and in the absence of other satisfactory treatment options.

This approval stems from efficacy and safety results obtained in recent studies including several solid tumours: ROAR (NCT02034110), NCI-MATCH (NCT02465060), and the CTMT212X2101 study (NCT02124772) in 36 paediatric patients.

The ROAR study included patients with high-grade glioma, biliary tract cancer, low-grade glioma, small bowel adenocarcinoma, gastrointestinal stromal tumour and anaplastic thyroid cancer. The NCI-MATCH trial included patients with BRAF V600E-positive solid tumours (excluding melanoma, thyroid carcinoma and colorectal carcinoma), while the paediatric trial included patients with refractory or recurrent low or high grade glioma. Overall, the objective response rate (ORR) was 41% among the 131 adult patients (95% CI, 33%-50%) [180–183].

The determination of tumor genomic profile requires analysis of tumour DNA by tissue biopsy. However, tumour biopsies, to date considered the gold standard in molecular tumour characterisation, have some important limitations. Liquid biopsy, on the other hand, is a non-invasive and easily repeatable diagnostic technique that can capture genomic heterogeneity within the patient and during therapy and represents a promising and innovative approach that could greatly facilitate access to agnostic therapies for more patients [1].

Although clinical biopsy overcomes some of the many limitations of standard tissue biopsy, it struggles to officially enter standard clinical practice. To date, liquid biopsy, using qPCR, has been approved by FDA and EMA for the detection of EGFR mutations in non-small cell lung cancer (NSCLC) and Kras mutations in colorectal cancer (CRC) [184–186]. Furthermore, liquid biopsy is recommended in the determination of resistance mechanisms in advanced NSCLC, in particular the T790M resistance mutation [187, 188].

Liquid biopsy has also shown promise in the agnostic indication of therapy, although still not officially approved and recommended by clinical practice guidelines compared to standard tissue biopsy.

Recently, the predictive value of TMB assessed on liquid biopsy (bTMB) was investigated in 2 different prospective studies. Both these studies showed that high TMB assessed on peripheral blood in patients with advanced NSCLC correlated with better outcomes during immunotherapy [155, 156]; in particular the phase 2 B-FIRST trial reported a greater overall response rate and a trend toward better Progression Free Survival (PFS) and Overall Survival (OS) in patients with high bTMB treated with atezolizumab.

However, the technique for determining TMB on peripheral blood is not yet standardised and therefore not officially recommended in clinical practice.

Tissue biopsy also remains the gold standard in the determination of MSI/dMMR, assessed by immunohistochemistry or molecular assays. However, liquid biopsy could also overcome important limitations in this field, especially intratumour heterogeneity, within the single disease site or between different disease sites (primary tumour and metastases) [189]. Indeed, the use of liquid biopsy could allow a rapid expansion of treatment options in patients with various solid tumours. A high degree of concordance between tissue-based MSI determination and MSI determination based on circulating tumour DNA has been reported in the literature [190, 191]. NGS is capable of analysing microsatellites at thousands of loci simultaneously and, at the same time, can assess the mutational profiling in targeted regions. It has been shown to determine both MSI and TMB status, achieving excellent sensitivity [192]. Among the NGS-based approaches, the Guardant360® CDx (Guardant Health, Redwood city, CA, USA) and the liquid CDx FoundationOne® (Foundation Medicine, Cambridge, MA, USA). Medicine, Cambridge, MA, USA) are FDA-approved blood-based diagnostic tests and are considered suitable for the determination of MSI on peripheral blood samples [157]. It has been shown that the Guardant360® CDx has an overall accuracy of 98.4% and a higher concordance between MSI on cell free DNA (cfDNA), tissue PCR and NGS than immunohistochemistry [158].

For the determination of NTRK rearrangements various tissue analysis techniques have been employed over the years, including NGS, immunochemestry and fluorescent in situ hybridization (FISH) [193].

The possibility of using liquid biopsy in the evaluation of NTRK fusions could ensure fast access to specific drugs for many patients, even in the case of insufficient or inadequate tumour tissue. Some plasma-based NGS have demonstrated in the literature a high degree of concordance with tissue genomic tests, although, actually, the potential of liquid biopsy in identifying NTRK fusions is largely unknown [159, 194].

Recently, a retrospective study reviewed ctDNA analysis data obtained with the Guardant360 cfDNA assay in patients with advanced solid tumours. The study showed that the presence of NTRK1 fusions in ctDNA was confirmed on tissue analysis in 88% of cases [160]. In view of the accessibility of two specific drugs for this molecular target, the potential of liquid biopsy should be explored in the detection of NTRK rearrangements to improve the identification of patients who may benefit from NTRK-specific treatments.

In light of the recent approval of Dabrafenib-Trametinib therapy in BRAF mutated neoplasms, liquid biopsy would represent an innovative approach that would also facilitate access to this treatment option for many neoplasms. However, most of the published literature on the clinical use of liquid biopsy to detect patients with BRAF mutation concerns maily mCRC, melanoma and NSCLC, while few data are available on less frequent types of cancer. Gonzales-Cao et al. reported the results of quantitative PCR analysis conducted in 92 serum and plasma samples from lung, colon and melanoma archives with paired tumour tissue, succeeding in detecting and quantifying BRAFV600E in mixed samples with a specificity of 100% and a sensitivity of 57.7% [161, 162]. Moreover, the RASANC study led to the approval of Idylla (Biocartis, Inc., Jersey City, NJ), a real time PCR-based assay for the detection of KRAS, NRAS and BRAF in metastatic colon cancer. The multicentre prospective study RASANC (NCT02502656), which included 98 patients with metastatic colon cancer, retrospectively assessed for the presence of ctDNA mutations in KRAS, NRAS and BRAF using the fully automated Idylla platform, showed an overall concordance between Idylla and NGS for BRAF of 99.5% [163, 164].

On the other hand, a recent systematic review comparing liquid biopsy and tissue biopsy with NGS analysis in NSCLC, showed that for BRAF mutation the positive percent agreement was inferior to 60%, probably due to the small size of cases [165]. Recently, in a small study it was possible to detect a BRAF V600E mutation in the plasma of 4/5 patients with BRAF V600E mutant brain tumors (both gliomas and brain metastasis) confirmed by ddPCR assay. Definitely, the method of analysis of Braf mutation in liquid biopsy would deserve further investigation in patients with different types of solid tumours [166].

The role of liquid biopsy has been extensively investigated in detecting PIK3CA-mutated breast tumors. Tumors carrying PIK3CA mutations may be sensitive to PIK3CA inhibitor drugs, although it is far from being considered a driver mutation proper. On 24 May 2019, the Food and Drug Administration approved alpelisib (PIQRAY, Novartis Pharmaceuticals Corporation) in combination with fulvestrant in metastatic/advanced, hormone receptor-positive, HER2-negative breast cancers carrying PI3CA mutation, after progression from a first-line endocrine therapy. The therascreen® PIK3CA RGQ PCR Kit diagnostic test, (QIAGEN Manchester, Ltd.), has also been approved to detect patients with PIK3CA mutations, which can be performed either on tumour tissue samples and/or in circulating tumour DNA (ctDNA) in plasma [195].

The phase 3 SOLAR-1 study led to the approval of this drug in breast cancer: median PFS was superior in the experimental arm, 11.0 months (95% CI: 7.5, 14.5) compared to 5.7 months (95% CI: 3.7, 7.4) in the control arm (HR 0.65; 95% CI: 0.50, 0.85; *p* = 0.001). In contrast, the median OS was 39.3 months (34.1–44.9) in the alpelisib-fulvestrant arm versus 31.4 months in patients of placebo-fulvestrant arm (*P* = 0.15) without reaching statistical significance, but, anyway, supporting the benefit of the combination in this PIK3CA-mutated patient population [195, 196]. In a phase Ia study (NCT01219699), alpelisib demonstrated tolerable safety and encouraging preliminary activity in patients with PIK3CA-mutant solid tumours, suggesting a rationale for its use alone or in combination with other drugs in the treatment of PIK3CA-mutant solid tumours [197].

A further Phase Ib, multicentre, open-label study recruited patients with advanced solid tumours and evaluated the combination of alpelisib and paclitaxel. Unfortunately, the safety profile was found to be of concern in patients with advanced solid tumours, and the study was terminated [198]. Further studies are needed to assess whether alpelisib may have an agnostic indication in solid tumours carrying the PI3KCA mutation.

Liquid biopsy represents an innovative approach that, in the era of agnostic therapies, would allow a rapid, minimally invasive and easily repeatable assessment of the genomic tumor profile. Further trials to validate and standardise analysis techniques in solid tumours are urgently needed to expand the use of liquid biopsy in clinical practice. Indeed, liquid biopsy could have a fundamental impact on a patient's oncological history in at least 2 situations: 1) at the time of diagnosis, in patients with insufficient tumour tissue for genomic profiling or inaccessibility of the tumour site to be biopsied 2) at the disease progression, to detect acquired resistance mechanisms. In both cases, an improved detection rate of molecular targets, eligible for agnostic therapies, could be achieved.

## Role of liquid biopsy in monitoring the dynamics of CGP during anticancer therapies: the role of genomic reprofiling

Despite the multiple applications of liquid biopsy Comprehensive Genome Profile (CGP), most of the evidence concern metastatic setting and in particular the analysis of ctDNA rather than CTC or extracellular vesicles whose results today would seem less informative [199]. Several experiences in the most burdening disease (CRC, BC, and NSCLC) attest to the high agreement (> 80%) [200–202] in genomic profiling through tissue or liquid biopsy [203–205] (Table 8). Among the numerous fields of application through the patient journey, CRC liquid biopsy application was conceived in primary anti EGFR moAbs primary resistance linked to mutant RAS and BRAF status. Hence, NGS retrospective analysis of 92 patients from the CAPRI-GOIM [206] study using tissue and liquid biopsy showed similar PFS and OS comparing K-RAS exon 2 WT and RAS mutant patients [207]. Liquid biopsy in a prospective trial was useful for predicting emerging resistance genetic variants on several genes during treatment with anti-EGFR MoAbs as well as better prognosis for those patients with circulating wild-type biomarkers [208], although results in a similar context from other trials such as the phase III ASPECCT [209] suggested a less severe prognosis for mCRC patients treated first with Panitumumab developing emerging circulating mutations in RAS/BRAF pathway. In this regard, considering RAS mutations, BEAMing liquid biopsy showed better diagnostic accuracy than the tissue one (BEAMing and NGS) in a small series including paired tissue and liquid samples to detect rising resistance mutations (57.1% vs 7.1% and 9.5%, respectively, *p* = 0.008) [210] suggesting its specific utility in highlighting subclones under selective pressure during treatments with anti-EGFR. Therefore, these consistent results have been investigated on other genes involved in growing resistance such as HER-2, BRAF, or MET [211–215]. Recently, as a matter of course, liquid biopsy profiling has been the rationale for the development of rechallenge strategies. CRICKET trial [216] constitutes the proof-of-concept study in this setting, although in a small series of patients. In particular, investigators enrolled tissue confirmed RAS/BRAF WT mCRC population in which, of the 28 patients studied with ctDNA, only RAS/BRAF WT achieved a partial response with a strategy of anti-EGFR reintroduction. The most recent biomarker-driven CHRONOS trial [217] has strengthened these results by proposing the rechallenge strategy only to RAS/BRAF WT patients achieving a RECIST response and at least a 50% reduction in RAS ctDNA mutant fraction before receiving anti-EGFR retreatment. Confirmatory data of the phase II CAPRI-2 study (NCT05312398) evaluating the rechallenge with Cetuximab plus Irinotecan in mCRC patients harboring a RAS/BRAF mutant status after a first-line anti-EGFR first-line regimen are awaited.

**Table 8:** Summary of the studies evaluating the cfDNA dynamics included in this paragraph

Trial	First author	Disease context	Intervention control groups	cfDNA technique	Gene investigated
CAPRI-GOIM	F. Ciardiello	mCRC	- FOLFOX + Cetuximab - FOLFOX	NGS	KRAS/NRAS/BRAF/PIK3CA
ASPECCT	TJ. Price	mCRC	- Panitumumab - Cetuximab	BEAMing NGS	KRAS
CRICKET	C. Cremolini	mCRC	- Cetuximab + Irinotecan	ddPCR NGS	RAS/BRAF
CHRONOS	A. Sartore-Bianchi	mCRC	- Panitumumab	ddPCR NGS	RAS/BRAF/EGFR
CAPRI-2	ongoing	mCRC	- Cetuximab - FOLFIRI - FOLFOX regimen - Irinotecan	NGS	RAS/BRAF
AURA-3	TS Mok	mNSCLC T790M +	- Osimertinib - Carboplatin + Pemetrexed	NGS	EGFR
FLAURA	SS Ramalingam	mNSCLC	- Osimertinib - Gefitinib - Erlotinib	NGS	EGFR
NILE	RD Page	mNSCLC	/	NGS	EGFR/ALK/ROS-1/BRAF MET/ERBB2/RET
BFAST	R Dziadziuszko	mNSCLC	- Alectinib	NGS	ALK
ALEX	TS Mok	mNSCLC	- Alectinib - Crizotinib	NGS	ALK
APPLE	J Remon	mNSCLC EGFR +	- Osimertinib - Gefitinib	NGS	EGFR
/	C Aggarwal	mNSCLC	NGS–indicated therapy	NGS	EGFR/ALK/MET/BRCA1/ROS1/RET/ERBB2/BRAF
PALOMA-3	O'Leary	mBC	- Palbociclib + Fulvestrant - Placebo + fulvestrant	NGS	RB1/ PIK3CA/ESR1
PLASMA-MATCH	NC Turner	mBC	- Fulvestrant - Neratinib - Capivasertib	NGS	AKT1/HER2/PTEN/ESR1

*Abbreviations*: *mCRC* metastatic colorectal cancer, *NGS* Next-generation sequencing, *FOLFOX* 5-fluorouracil/leucovorin combined with oxaliplatin, *KRAS* Kirsten RAt Sarcoma virus, *NRAS* Neuroblastoma ras viral oncogene, *BRAF* v-raf murine sarcoma viral oncogene, *PIK3CA* Phosphatidylinositol-4,5-Bisphosphate 3-Kinase Catalytic Subunit Alpha, *BEAMing* Beads, emulsion, amplification, magnetics, *ddPCR* droplet digital polymerase chain reaction, *EGFR* Epidermal growth factor receptor, *mNSCLC* metastatic Non-Small Cell Lung Cancer, *ALK* Anaplastic lymphoma kinase, *ROS-1* ROS Proto-Oncogene 1, *MET* Mesenchymal Epithelial Transition, *ERBB2* Erythroblastic oncogene B, *RET* Rearranged during transfection, *mBC* metastatic breast cancer, *RB1* Retinoblastoma protein 1, *ESR1* Estrogen Receptor 1, *AKT1* AKT serine/threonine kinase 1, *HER2* Human epidermal growth factor receptor 2, *PTEN* Phosphatase and tensin homolog

In the last two decades, oncogene-addicted NSCLC patients did experience a therapeutic revolution linked to the introduction of tyrosine kinase inhibitors (TKIs) and their combinations aiming to overcome primary and secondary resistance growing up [218]. However, this scenario is rapidly changing due to emerging resistance (on-target, off-target bypass pathways, and histological transformations) [219–222] following treatment with 3rd generation EGFR-TKI as a second or first-line option following the results of AURA-3 [223] and FLAURA trials in mNSCLC patients carrying an EGFR sensitizing mutation. In this way, several new drugs have been tested in combination with upfront Osimertinib to overcome acquired resistance, mainly due to -MET (about 15%) genomic alterations. As for EGFR inhibitor TKIs, studies with ALK-TKIs demonstrate a profound variety of resistance mechanisms [224–226] which differ according to I, II, or III generation molecules. In particular, Shaw et al. [225] showed that the use of Lorlatinib, a 3rd generation ALK-TKI, produced almost identical ORR when evaluated in tissue or plasma (69% vs 62%) samples. However, several factors can undermine the diagnostic accuracy of liquid biopsy CGP affecting ctDNA levels. On the one hand, biological and pathological factors, such as tumor burden, anatomical site (intrathoracic vs extrathoracic), histology (adenocarcinoma vs squamous), proliferative index, necrosis, and the type of fluid investigated [36, 227]; on the other hand, a series of scientific shreds of evidence have shown that quite resistances to TKIs, not only EGFR-linked, are polyclonal and monoclonal and this would affect the disease biological evolution among different patients [228, 229]. In recent years, international scientific societies receipt liquid biopsy and NGS profiling as useful tools to provide clinically valuable information throughout the patient's therapeutic pathway [52, 53, 230, 231] to be included as a complementary opportunity for tissue biopsy. In the NILE study [232], although only 18% of patients received complete genotyping across the 8 advanced NSCLC guideline recommended biomarkers, liquid biopsy genomic profiling on 282 increased sensitivity (80%) for any of them. Interestingly, for EGFR, ALK, ROS-1, and BRAF the concordance and positive predictive value rates of tissue-plasma analyses were 98.2% and 100%, respectively. Furthermore, LB profiling increased the tissue diagnostic ability by about 48% with also a turnaround time (9 vs 15 days) benefit, supporting a plasma-first approach. Similarly, the phase II/III BFAST study [34, 100, 233] in the ALK + naïve cohort recently showed an intriguing high ORR (87.45 by INV and 92% by IRF) to ALK-targeted therapy after blood-based testing, when compared with data from the ALEX study (71.7%) [234]. These results can be explained by the inability of the tissue analysis to overcome issues related to both intratumoral and intrapatient heterogeneity. Likewise, data showed various limits of tissue biopsy to capture the subclonal population of tumor cells with distinct alterations as well as to intercept the single lesion-specific alterations [235]. Remarkably, not all patients are susceptible to new tissue sampling for disease reprofiling. In this regard, Remon J et al. in the APPLE trial (ESMO Annual Congress 2022) support the serial monitoring of the T790M mutation through LB sampling in a cohort of advanced NSCLC patients undergoing upfront gefitinib and Osimertinib. In particular, preliminary results of arm B (plasma-guided GefitinibrOsimertinib sequence) versus arm C (imaging-guided GefitinibOsimertinib sequence) underline that LB can detect a biochemical progression before radiological evaluation in 17% of cases with a 10% improvement in 18-month interim OS rate benefit (87% vs 77%). Although the analysis of ctDNA poses numerous challenges related to its highly variable fraction, fragmentation, and half-life, Aggrawal C. et al. (30325992) demonstrated in a prospective cohort sub-analysis of 67 NSCLC mNSCLC patients investigated with a 73-gene NGS platform that plasma-based biomarkers with low-allele frequency may respond to targeted therapy by achieving an overall disease control rate of 85.7%. Liquid biopsy CGP could also provide an important contribution to understanding the kinetics of the antitumor response. In this context, Mack PC et al. showed that EGFR ctDNA clearance after 60 days of EGFR-TKI and anti-EGFR-MoAb combination regimen correlated with substantial improvement in PFS and OS in a cohort of advanced NSCLC underwent a 73-gene blood-based NGS panel suggesting a role of LB in determining novel pharmacodynamic predictive biomarkers of response/resistance to targeted agents [236].

Emerging data support the use of genomic profiling by LB also in breast cancer both to determine the emergence of resistance and for dynamic monitoring during therapy, in particular, those based on hormone therapy. An analysis of the phase III PALOMA-3 study by O'Leary et al. [237, 238], comparing the combination of Fulvestrant + Palbociclib vs Fulvestrant + Placebo, 14 patients underwent paired ctDNA exome analysis showing biological signs of clonal evolution in 85% of cases with new emerging mutations both in all cohorts (PIK3CA, ESR1) or only in the Palbociclib combination arm (RB1) emphasizing a subclonal complexity of hormone-responsive breast cancer. In particular, the ESR1 Y537S mutation appears to be the major driver of resistance to Fulvestrant. The phase 2a PLASMA-MATCH platform multiarm study [239] showed the opportunity of ctDNA testing to select patients for a personalized approach. In this study, Turner NC et al. did enroll advanced breast cancer patients already treated with >  = 2 hormone therapy options to perform a plasma-based NGS analysis to be divided into 4 parallel treatment groups according to mutational status (ESR1 mutations, HER2 mutations; AKT1 mutations and estrogen receptor-positive; AKT1 mutations and estrogen receptor-negative or PTEN mutation) in order to receive a tailored plasma-guided treatment. Results confirm a sufficient number of objective responses in cohorts B (HER-2 mutation, 5/20) and C (AKT1/ER + , 4/18) to further explore this scheme supporting its inclusion in future clinical practice. This evidence, bearing the polyclonal heterogeneity toward ER + breast cancer evolution, attests to the potential benefit of liquid biopsy CGP to capture different disease progression patterns expressing both polyclonal ESR1 and MAPK mutations significantly affecting survival outcomes or to distinguish between clonally dominant or sub-clonal variants [240] helping in the interpretation of tumor heterogeneity through the creation of genomic signatures related to the different histological profiles of breast cancer. Besides, the level of ctDNA in the plasma should be potentially useful for the monitoring of disease. A close relationship has been highlighted between ORR and the decrease/increase of ctDNA levels during disease response/progression [241] offering the opportunity to optimize treatment customization using combinatory regimens. This is supported by recent evidence that demonstrates the importance of testing the early ctDNA dynamics to select patients who underwent rapid disease progression [238, 241]. What remains to be established is the best time to optimize a liquid biopsy CGP approach [242] during the disease as well as interventional studies focused on catching plasma-based early dynamic changes.

## Role of liquid biopsy in immunotherapy: limits and perspectives

Despite the durable, long-lasting responses for some patients with advanced solid tumors, the clinical benefit of Immune-checkpoint inhibitors (ICIs) is still limited to selected patients, as a result of primary or acquired resistance to therapy [243].

One of the major challenges in the field of cancer immunotherapy is the development of a robust and dynamic predictive biomarker for optimal patient selection [244]. These extensive efforts in biomarker research have led to biomarker-based, tissue-agnostic, approvals of ICIs for the treatment of patients whose tumors harbor microsatellite instability (MSI) or high tumor mutation burden (TMB) [245]. However, the currently available biomarkers, often rely on tumor tissue samples, such as elevated tumor PD-L1 expression in the tumor microenvironment (TME) [246, 247], the tissue TMB (tTMB) [248, 249], and others, have been unable to accurately identify the subset of cancer patients who benefit from these therapies. The plastic, dynamic, and multifactorial interaction of the tumor and host immune system under immunotherapy, makes the response to ICIs and its prediction a complex and winding process.

Following the promising results in targeted therapies, an increasing number of clinical studies are investigating the potential use of liquid biopsy to improve our ability to select the patients who are likely to respond to immunotherapy-based therapy [250] (Table 9).

**Table 9:** Summary of innovative applications of liquid biopsy and key studies in the context of immunotherapy

Localized Disease				
	**Type of analysis**	**Study**	**ICI Treatment**	**Tumor**
**Neoadjuvant ICI: Stratification/early assessment of efficacy**	ctDNA MRD	**CheckMate-816 trial** Forde PM, 2022 [251]	Nivolumab + platinum-based CT or platinum-based CT alone, followed by resection	NSCLC
**Adjuvant ICI: Stratification/early assessment of disease recurrence**	ctDNA MRD	**IMvigor010 trial** Powles T, 2021 [149]	Atezolizumab vs observation	Urothelial carcinoma
		**IMpower010 study (exploratory analyses)** Felip E., 2022 [252]	CT followed by atezolizumab vs best supportive care	NSCLC
**Advanced/Metastatic Diseased**				
**Treatment selection**	Baseline bTMB	**CheckMate 848** He et al., 2022 Schenker et al., 2022 [253, 254]	Nivolumab + ipilimumab vs nivolumab monotherapy	Pan-cancer
		**B-F1RST** Kim et al., 2022 [156]	Atezolizumab	NSCLC
		**BFAST** Peters et al., 2022 [252]	Atezolizumab vs chemotherapy	NSCLC
		**NEPTUNE** de Castro Jr et al., 2022 [255]	Durvalumab and tremelimumab vs chemotherapy	NSCLC
		**MYSTIC** Si et al., 2021 [256]	Durvalumab and tremelimumab vs chemotherapy	NSCLC
		Wang et al., 2019 [257]	Anti-PD-1/PD-L1	NSCLC
		**OAK/POPLAR** Gandara et al., 2018 [155]	Atezolizumab vs docetaxel	NSCLC
		**Khagi et al., 2017** [258]	Anti-PD1/PDL1/CTLA4	Pan-cancer
**Treatment selection**	Baseline bMSI	Georgiadis A, 2019 [259]	PD-1 Blockade	Pan-cancer
		Willis J, 2019 [158]	Immune Checkpoint Blockade	Pan-cancer
		**KEYNOTE-016 study** Le DT, 2015 [167]	Pembrolizumab	Colorectal/not colorectal cancers
**Early monitoring of response/resistance to ICI**	ctDNA longitudinal monitoring	Bratman SV, 2020 [260]	Pembrolizumab	Pan-cancer
		Váraljai R, 2020 [261]	Immune Checkpoint Blockade/Targeted Therapy	Melanoma
		Guibert N, 2019 [262]	Immune Checkpoint Blockade	NSCLC
		Goldberg SB, 2018 [263]	Immune Checkpoint Blockade	NSCLC
		Kim ST, 2018 [264]	PD-1 Blockade	Gastric Cancer

*CT* Chemotherapy, *MRD* Minimal Residual Disease, *NSCLC* Non-small cell lung cancer

Liquid biopsy is emerging as a minimally invasive, cost-effective and dynamic approach to assessing the landscape of intratumoral heterogeneity and longitudinal tumor evolution during ICI treatment [245]. Different targets were actively studied using liquid biopsy. Some examples are the evaluation of PD-L1 expression on Circulating Tumor Cells (CTCs) [265–267], the T-cell receptor (TCR) repertoire isolated from patients’ blood [268–270], and the circulating plasma or serum proteins, such as the soluble PD-L1 and PD-1 [271]. Recent findings indicate that the soluble forms of immune checkpoints can be detected in the peripheral blood [272, 273] and a correlation between baseline concentrations with clinical response was recently described in several cancer types [274–276]. However, the cell-free DNA (cfDNA), and their tumor-derived fraction (ctDNA), are currently the most advanced and studied approaches to liquid biopsy in the context of cancer immunotherapy. Particularly, the global quantification and kinetics of cfDNA/ctDNA during ICI treatment in the metastatic setting, the ctDNA-based assessment of blood TMB (bTMB) and blood MSI (bMSI) are mostly explored for patient selection [277].

The blood-based analysis of TMB and its role as a predictive biomarker of ICI response was retrospectively investigated in several clinical trials with promising findings. The POPLAR, OAK and MYSTIC trials included patients with metastatic NSCLC [155, 256]. In patients treated with atezolizumab versus docetaxel within the POPLAR and OAK trials, a high bTMB with a TMB cut-off of 16 mutations/Mb was associated with improved PFS and OS [155]. Subsequently, in the MYSTIC trial, that compared durvalumab and tremelimumab versus chemotherapy, a high bTMB (bTMB > 20 mutations/Mb) showed improved clinical outcomes [256]. Despite the promising results in retrospective trials, prospective studies in NSCLC have not confirmed the utility of bTMB to predict ICI response. In the phase 2 B-F1RST trial of atezolizumab monotherapy [156], and the phase 3 BFAST Trial of atezolizumab versus chemotherapy [252], a high bTMB using predefined bTMB thresholds of > 16 mutations/Mb, showed an increased ORR, further improved with higher bTMB thresholds. However, significant differences in PFS between high and low bTMB patients were not shown. Similarly, in the phase 3 NEPTUNE trial of durvalumab and tremelimumab versus chemotherapy, bTMB > 20 mutations/Mb fails to predict a clinical benefit [255].

Similar to bTMB, the predictive value of blood MSI has been investigated in patients treated with ICIs, using panel NGS or droplet digital PCR. The blood-based assessment of MSI, detected in ctDNA, was highly concordant with tissue-based testing and in predicted PFS in patients treated with ICIs [158, 167, 259]. However, the predictive role of bMSI for ICI therapeutic response has not been adequately investigated in prospective studies. For this reason, additional analyses and prospective validation are required to further explore the validity of bMSI for determining tumor MSI status and its predictive value.

Other potential innovative applications of liquid biopsy in the context of immunotherapy are the minimal residual disease (MRD) detection in the adjuvant/neoadjuvant setting, and the longitudinal response monitoring through ctDNA assessment during ICI treatment in the metastatic disease [245]. In the postoperative setting, ctDNA-based MRD detection may provide a useful tool to identify high-risk patients and to adequately select the subgroup for adjuvant treatment. To date, the utility of post-operative ctDNA detection is under investigation in several studies. In the IMvigor010 trial on urothelial carcinoma, the ctDNA detection after surgery showed improved outcomes in terms of disease-free survival (DFS) and OS in the atezolizumab group compared to the observation group of patients [149].

In the neoadjuvant setting, the association between ctDNA clearance and tumor response has been explored in patients with NSCLC [278]. In phase 3 CheckMate-816 trial, the patients with stage IB to IIIA resectable NSCLC were treated with nivolumab plus platinum-based chemotherapy or platinum-based chemotherapy alone, followed by resection [278]. Although a prospective validation is warranted, the data suggest that the pretreatment levels of ctDNA and the clearance during neoadjuvant treatment may be an early predictor of disease relapse after surgery [278].

Finally, in addition to the pre-treatment assessment of ctDNA as a predictive factor of ICI response, the longitudinal monitoring of ctDNA dynamics as an early predictor of tumor responsiveness is an area of active clinical research in the metastatic setting. Several studies support the ctDNA dynamic detection during the ICI treatment, highlighting how the “on-treatment” increased ctDNA levels is often related to progressive disease. On the other hand we have to consider that plasma genotyping demonstrated negative prognostic value of TP53 mutations appearance and negative predictive value of KRAS/STK11 and KRAS/STK11/TP53 co-mutations. Moreover, another potential source of false positive results is the possible contamination of hematopoietic or smoke-induced mutation that could compromise the predictive value of TMB count in liquid biopsy [261–263, 279].

Currently, advancing technologies and the recent promising clinical data in the era of immunotherapy make liquid biopsy a rapidly evolving field. However, several barriers still limit the transfer of liquid biopsy into clinical practice. Beyond the known analytical and clinical validation framework, and the clinical need for a perspective and robust validation of findings, one major challenge exists.

Anticancer immunity is a dynamic, complex, and context-dependent process. Thus, the plasticity of the immune system under immunotherapy, makes limited the validity of liquid biopsy when a single target is studied. Probably, only combinatorial strategies will able to capture the complexity of the continuously evolving tumor immune microenvironment, to precisely predict the response or resistance to immunotherapy [280].

## Role of liquid biopsy in analyses of vesicular genome

Over the last decade, apart from ctDNA, other members of the growing liquid biopsy “family” such as EVs or specific subtypes of EVs (namely, exosomes), have increasingly aroused considerable interest as a valuable biosource of cancer biomarkers [281–284].

From a historical perspective, EVs used to be considered lipid-rich particles isolated from cell culture supernatants and physiological fluids while only serving as disposal of cellular waste products [285]. To date, a growing body of evidence defined EVs as nanoscale-sized particles that, even if released under physiological and pathological conditions in the body fluids from almost all living lipid bilayer cells, seemed to be involved in cell-to-cell communication, promoting cross-talk between cancer cells within the tumor microenvironment while mediating tumor response and progression [286]. In this vein, emerging preclinical and clinical data supported the investigation of their use as either a compelling diagnostic tool or even a delivery approach for therapeutic purposes [287].

Following the minimal requirements released by the International Society of Extracellular vesicles (ISEV), EVs should be subclassified according to physical characteristics (size and density), biochemical composition, descriptions of conditions or cell of origin [288]. Although the biogenesis pathway remains far from clear with no wide consensus established yet, it is acknowledged that exosomes seemed to be generated by the fusion of multivesicular bodies in the late endosome whereas larger microparticles/microvesicles revealed to share a plasma membrane-derived origin [289]. The exploration of such EVs has increasingly been implemented in the cancer research field owing to their cell-specific cargo containing either proteins or nucleic acids, playing a crucial role in the intercellular exchange of genetic information [290].

Although being recovered from different other biofluids, the preferred source for EV isolation is blood plasma since serum might harbor further EVs additionally released during the clot formation [291, 292]. Compared to ctDNA and circulating tumor cells (CTCs), the presence of large and stable amounts of circulating EVs certainly represent major advantages, despite the high variability in diagnostic assays and clinical datasets [293]. Independently from the underlying mechanism of origin, EVs could be numerically easier to obtain than CTCs [294] while being more stable and representative than ctDNA in depicting the parental biological cargo [295].

Besides enclosing both protein-coding and non-coding RNAs, EVs also express proteins on their surface that proved to be useful for prognostication and therapy monitoring, supporting the clinical implementation of these analytes as relevant carriers of tumor genome in different cancer settings [296]. In this vein, plasma EV-associated molecules (such as DNA and non-coding RNAs) and proteins (mainly PD-L1) have been widely investigated as biomarkers for predicting therapeutic response [297]. Namely, in patients with advanced NSCLC undergoing immunotherapy, dynamic changes of plasma EV PD-L1 were significantly associated with survival, recently underlining even a better prediction for durable response than tissue PD-L1 [298]. Likewise, EV-associated miRNAs and long non-coding RNAs have received global attention in the longitudinal monitoring of systemic treatments in melanoma, breast and prostate cancer [299–301]. Further, a dynamic increase in plasma-derived EV *KRAS* or *EGFR* mutations seemed to be reliably suggestive of disease progression in pancreatic and lung cancer, respectively [302, 303].

However, the lack of harmonization of the different isolation and characterization techniques along with the low purity of circulating tumor-derived EVs critically affected the broader use of such promising biomarkers for functional research, further limiting the future implementation in the clinical practice [288, 304].

In this fascinating scenario, a multi-omic strategy combining EV information on either the DNA, RNA or protein level with the other liquid biopsy analytes might more comprehensively inform the molecular profile of patients with cancer while tailoring the most personalized therapeutic approach.

## Role of liquid biospy in analyses of other biological fluids (saliva, urine, fecal)

Although the majority of liquid biopsy research has focused on blood- based biomarkers, a plethoraof alternative sources of cancer-derived molecules such as circulating tumor DNA (ctDNA) and circulating microRNAs are now emerging [305–307]. In this section, we discuss existing evidence supporting the utility of analyzing non-blood biological fluids including urine, saliva and stool to identify potential diagnostic, prognostic and predictive biomarkers.

### Role of liquid biopsy in urinary samples

Several evidence suggested the potential clinical use of urine as a source of liquid biopsy for cancer diagnosis, disease monitoring and prediction of relapse (Table 10). ctDNA represents the most promising biomarkers in urine sample. It comprises of two distinct fractions: transrenal tumour DNA (trtDNA), which originates from plasma and enters the urine through glomerular filtration; urinary cell- free DNA (ucfDNA) which derives from cells shedding directly from the urinary tract [308]. trtDNA is, therefore, limited in size (typically < 250 bp) by virtue of undergoing renal filtration, while ucfDNA can be of larger molecular weight.

**Table 10:** Summary of findings about the role of liquid biopsy in urine samples

Type of marker	Type of tumor	Study endpoint	Findings	Reference
trtDNA	NSLC	Analysis of EGFR mutation status	High concordance of EGFR mutation status between urine, plasma and tissue; combined analysis of urinary and plasma ctDNA improved the detection of all T790M mutations compared with those detected with tissue-only	Reckamp et al. 2016 [17]
trtDNA	NSLC	Analysis of KRAS status	High concordance of KRAS mutation status between urine and tissue	Wang 2017; Xie 2018 [309–311]
trtDNA	CRC	Analysis of KRAS and BRAF mutation status	High concordance of EGFR mutation status between urine, plasma and tissue	Yu 2019 [312]
trtDNA	CRC	Early diagnosis	trtDNA is a high sensitive method for early cancer detection	Tian 2017 [313]
trtDNA	Breast cancer	Disease monitoring and prediction of relapse	Longitudinal analysis of trtDNA concentration is a sensitive method for the monitoring of disease and the prediction of relapse in early stage disease	Zuo 2020 [314]
trtDNA	Hepatocellular carcinoma	Prediction of relapse	trtDNA has been detected prior to radiological evidence of disease recurrence	Hann 2017 [233]
ucfDNA	Bladder cancer	Early diagnosis	ucfDNA showed a great diagnostic potential for identifying cancer from hematuria patients	Zhang 2021 [315]
ucfDNA	Urothelial carcinoma	Early diagnosis	ucfDNA is a sensitive diagnostic method for identifying cancer-associated genomic alterations in patients with suspected urothelial carcinoma	Oto 2019; Springer 2018; Dudley 2019 [316–318]
ucfDNA	RCC	Early diagnosis	The analysis of ucfDNA methylome showed a high level of sensitivity in early cancer detection	Nuzzo 2020 [319]
ucfDNA	Prostate cancer	Early diagnosis	ucfDNA is a high sensitive method for early cancer detection	Casadio 2013 [320]
lncRNA	Prostate cancer	Early diagnosis and prognosis	Specific urinary lncRNAs, provide diagnostic and prognostic information better than PSA	McKiernan 2016; Sanguedolce 2016; Groskopf 2006; Whitman 2008 [321–324]
Exosomal RNA	Prostate cancer	Early diagnosis	The expression of specific exosomal RNA showed a high sensitivity in early cancer detection	Tutrone 2020 [325]
mRNA	Prostate cancer	Early diagnosis	The overexpression of two mRNA, DLX1 and HOXC6, provide diagnostic information	Hendriks 2021 [326]
lncRNA	Bladder cancer	Early diagnosis	lncRNAs are useful biomarker for early cancer detection	Srivastava 2014; Wang 2017 [309, 311, 327]

*trtDNA* transrenal tumour DNA, *ucfDNA* urinary cell- free DNA, *lncRNA* long-non-coding RNA, *mRNA* messanger-RNA, *miRNA* micro-RNA, *NSCLC* Non-Small Cell Lung Cancer, *CRC* colorectal cancer, *RCC* Renal Cell Carcinoma

The potential clinical use of trtDNA was mainly investigated in patients with non- small- cell lung cancer (NSCLC), testing for alterations in EGFR, including the T790M mutation and KRAS [17, 309, 328]. In a cohort of 63 patients from the Tiger- X trial the analysis of trtDNA demonstrated a detection sensitivity of EGFR specific mutations similar to that observed in plasma and tissue providing the early evidence of concordance between trtDNA and tissue EGFR status [17]. Interesting, combined analysis of urinary and plasma ctDNA improved the detection of all T790M mutations compared with those detected with tissue-only, suggesting a potential synergistic effect of combining different liquid biopsy methods [17]. Similarly, mutant KRAS DNA within urine specimens and primary tissue biopsies showed high levels of concordance [309, 310]. The potential clinical utility of trtDNA analysis is emerged also in colorectal cancer (CRC) where KRAS and BRAF mutation profile detected in urine overlapped with matched tumor tissue and plasma [312]. Liquid biopsy of trtDNA proved to be a high sensitive early detection method in CRC, breast and hepatocellular cancer [233, 313, 314, 329]. Indeed, KRAS mutations have been detected in urine samples of patients with stage I CRC despite the lower levels of ctDNA in the early disease [313]. Moreover, a longitudinal analysis of trtDNA concentration in early breast cancer patients showed that it could be a sensitive method for the monitoring of disease and the prediction of relapse [314]. Similarly, in patients with hepatocellular carcinoma, trtDNA has been detected prior to radiological evidence of disease recurrence suggesting its potential utility to complement imaging technique [233].

Tumors that occur within the urinary tract such as bladder, prostate and renal cell cancers (RCC) can release DNA fragments directly into urine as ucfDNA. A pilot study of bladder cancer patients demonstrated that specific gene mutation panel in urine had a great diagnostic potential for identifying cancer from hematuria patients [315]. Similarly, ucfDNA resulted a diagnostic method more sensitive than ctDNA in identifying cancer-associated genomic alterations in patients with suspected urothelial carcinoma [316]. Based on these promising results, a specific multiplex PCR- based assay, UroSEEK, has been developed for the early detection of urothelial carcinoma. In 570 patients at risk for bladder cancer, UroSEEK alone identified 83% of patients who went on to be diagnosed with bladder cancer, this sensitivity increasing to 95% when combined with urinary cytology [317]. Another recent high-throughput sequencing method for detection of urine tumor, called CAPP-Seq, proved to be not only a promising method of early cancer detection but also for monitoring disease progression or recurrence in patients with urothelial carcinomas [318]. Studies in RCC patients found less ctDNA than other tumour types and limited overlap between the plasma and urine ctDNA content [330]. However, the analysis of DNA methylome of ucfDNA showed a high level of sensitivity in early RCC detection [319]. A pilot study in prostate cancer and healthy volunteers revealed that ucfDNA might provide a more accurate alternative to serum prostate-specific antigen (PSA) for the early diagnosis of cancer [320]. Moreover, emerging evidence reported that urine could be a sensitive tool for the study of prostate cancer epigenetic alterations [331].

In summary, urinary ctDNA analysis provides results that are highly concordant and potentially complementary to those obtained from tissue and plasma ctDNA sequencing. In addition, the concentration of ctDNA in urine is higher than ctDNA from plasma since it derived from renal cell and urothelial carcinomas that occur within the urinary tract. Thus, in these tumors, the sensitivity of urinary ctDNA in cancer detection and/or recurrence is often greater compared to blood ctDNA and tumor tissue. Despite these advantages, certain critical issues in urinary ctDNA tests are emerged reducing their clinical development. Firstly, trtDNA content is limited by glomerular filtration and the rate of filtration can be highly variable and influenced by anticancer therapy. Secondly, ctDNA yield can vary by time since previous void: for example lower trtDNA yields are obtained from samples < 1.5 h after a previous void [332]. Finally, the methods of preservation and analysis of urinary ctDNA are not yet standardized and require further implementations.

In addition to urinary ctDNA, mRNA [264, 333], long non coding RNA (lncRNA) [334], miRNAs, PIWI-interacting RNA (piRNA) [335], and circular RNAs (circRNAs) [336], have been identified in urine samples as potential biomarkers in urological cancers. In particular, specific urinary lncRNAs, such as Prostate Cancer Antigen (PCA3), provide diagnostic and prognostic information better than PSA [321–324]. In this regards, Intelliscore test, a commercial exosome-based assay, was included in the National Comprehensive Cancer Network (NCCN) guidelines for prostate cancer early detection [325]. Another test, SelctMDx, based on the overexpression of two mRNA, DLX1 (distal-less homeo-box 1), HOXC6 (homeo-box C6), was recently developed as diagnostic tool in prostate cancer [326]. Urinary lncRNA proved to be useful biomarker also for bladder cancer detection [311, 327].

The most advantage of urinary liquid biopsy is the nature entirely noninvasively of samples that can be obtained within the patient’s home, without the need for venesection or the presence of health-care professional specialists. In addition, the entirely non-invasive sampling enables longitudinal analysis at different timepoints, without the need for hospital visits providing a unique benefit for patients with urological cancers. Moreover, urine can be collected in large volumes, which solves one of the major problems with tissue or blood-based liquid biopsy that are often limited by the quantity and the number of samples.

### Role of liquid biopsy in salivary samples

Saliva contains cells, proteins and nucleic acids and represents an alternative source of liquid biopsy [337]. Similar to urinary ctDNA, salivary ctDNA (sctDNA) mainly originates from local tumors such as head and neck squamous cell carcinomas (HNSCC), but it can derive also from distant malignancies through the blood across the mucosal membrane [338]. Several evidence reported that sctDNA could be a useful diagnostic tool for identifying patients with HNSCC [339] (Table 11). In oropharyngeal squamous cell carcinoma (OSSC), combined analysis of HPV-16 in plasma and saliva increased the sensitivity of identifying HPV-16–positive patients. Interesting, HPV DNA presence and concentration in saliva were correlated with disease recurrence and survival [340]. Similarly, in another study, salivary HPV DNA was correlated with tumor burden and predictive of treatment response [341]. A study that pooled different HNSCC tumor types showed that sctDNA is ideal for the assessment of the oral cavity cancers, while the combination analysis of plasma and saliva ctDNA is necessary to increase the sensitivity for diagnosis and prognosis of oropharynx, hypopharynx and larynx tumors [18]. The role of saliva-based liquid biopsy was also investigated in NSCLC, where a high concordance of EGFR mutations was found in sctDNA and plasma ctDNA [342]. However, saliva might not be a suitable sample for NSCLC diagnostics due to the low ctDNA concentrations entering the saliva from plasma [342]. In addition, sctDNA fragments are ultrashort (40–60 bp), thus conventional PCR techniques failed in assessing EGFR mutations in saliva. In this regard, novel and more sensitive technologies, such as the electric field- induced release and measurement (EFIRM) assay, are developed to detect EGFR alterations in sctDNA [343–345]. Currently, it represents the optimal method to analyze saliva samples from patients with malignancies other than HNSCC [346].

**Table 11:** Summary of findings about the role of liquid biopsy in saliva samples

Type of marker	Type of tumor	Study endpoint	Findings	Reference
sctDNA	HNSCC	Early diagnosis	sctDNA is a useful diagnostic tool for early cancer detection	Sethi 2009 [339]
sctDNA	OSSC	Disease recurrence and survival	HPV DNA presence and concentration in saliva correlated with disease recurrence and survival	Ahn 2014 [340]
sctDNA	OSSC	Prediction of treatment response	Salivary HPV DNA was predictive of treatment response	Hanna 2019 [341]
sctDNA	HNSCC	Early diagnosis and prognosis for oral cavity cancers;	Saliva ctDNA increaseS the sensitivity for diagnosis and prognosis of oropharynx, hypopharynx and larynx tumors	Wang 2015 [18]
sctDNA	NSLC	Analysis of EGFR mutation status	High concordance of EGFR mutation status between saliva and plasma	Ding 2019 [342]
mRNA	HNSCC	Early diagnosis	The expression of specific mRNAs showed a high sensitivity in early cancer detection	Li 2004; Elashoff 2012; Bu 2015; Chai 2016 [347–350]
miRNA	HNSCC	Early diagnosis and prediction of treatment response	The expression of specific miRNAs showed a high sensitivity in early cancer detection and is predictive of treatment response	Han 2018; Zahran 2015; Wu 2019; Uma 2020; Greither 2017; Ahmad 2019 [351–355]
LncRNAs; circRNAs	HNSCC	Early diagnosis and prognosis	LncRNAs and circRNAs showed a potential diagnostic and prognostic value	Tang 2013; Bahn 2014; Zhao 2018 [85, 227, 356–359]
Exosomal small RNA	Esophageal squamous cell carcinoma	Early diagnosis and prognosis	Saliva-derived exosomal small RNA signature provide diagnostic and prognostic information	Li 2022 [85, 227, 359]

*LncRNA* Long-non-coding RNA, *mRNA* messager-RNA, *miRNA* micro-RNA, *circRNA* circular RNA, *NSCLC* Non-Small Cell Lung Cancer, *HNSCC* Head and neck squamous cell carcinomas, *OSSC* Oropharyngeal squamous cell carcinoma

In addition to ctDNA, the potential diagnostic and prognostic role of salivary circulating tumor RNA (ctRNA) has also been investigated in HNSCC patients [360]. Interesting, several studies showed that salivary mRNA might be a potential biomarker for early detection and prognosis in HNSCC [347–350]. Similarly, specific salivary miRNA signatures were found in HNSCC patients suggesting their potential use in early detection [351–353]; other studies demonstrated the utility of saliva miRNAs as biomarkers also in predicting therapeutic response [354, 355, 361]. LncRNAs and circRNAs also showed a potential diagnostic and prognostic value in HNSCC [356–358], but additional research are needed to confirm these results. A recent multicenter study identified a saliva-derived exosomal small RNA signature for esophageal squamous cell carcinoma diagnosis, prognosis, and particularly, prediction of response to adjuvant therapy [359]. Finally, recent studies have been linked non-genome-based markers with the OSCC occurrence such as salivary metabolites and oral microbiome [362, 363].

In summary, similar to urine sample, saliva is another body fluid that can be non- invasively obtained without restrictions on sampling location and without the presence of a health-care professional. The ease of sampling enables longitudinal evaluation at multiple timepoints useful for monitoring treatment response and disease recurrence. In addition, sctDNA demonstrated to be a suitable diagnostic and prognostic tool in cancers of the oral cavity, while it provides useful information in combination with other techniques for assessing tumors of the oropharynx, hypopharynx and larynx. The major disadvantages are the low ctDNA concentrations and the limited fragment size that requires more advanced detection technologies such as EFIRM platform. Regarding salivary ctRNA, the main limitation is the risk of RNA degradation due to the presence of RNases in the saliva that could increase the false-positive and false-negative detection rates.

### Role of liquid biopsy in stool samples

The role of stool DNA as diagnostic biomarker for CRC is currently under investigation based on the evidence that early-stage colorectal lesions develop predominantly within the mucosa with epithelial shedding of DNA into the lumen of the colon (Table 12). In particular, a fecal DNA panel consisted of 21 mutations in KRAS, adenomatous polyposis coli and p53 tumor-suppressor genes showed a high sensitivity for detection of CRC compared to fecal immunochemistry and occult blood testing [364]. These promising results led to the development and approval by FDA of the first stool-based colorectal screening test (Cologuard) that detects the presence of specific cancer-associated DNA mutations [365, 366]. Although this assay is more sensitive compared to a commonly used occult blood testing, this technique is less cost- effective than the alternatives and might not be applicable for large- scale screening programs [367].

**Table 12:** Summary of findings about the role of liquid biopsy in stool samples

Type of marker	Type of tumor	Study endpoint	Findings	Reference
DNA	CRC	Early diagnosis	Fecal DNA mutation panel showed a high sensitivity for early cancer detection compared to fecal immunochemistry and occult blood testing	Imperiale 2009; Prince 2017; Redwood 2016 [364–366]
DNA	Pancreatic cancer	Analysis of KRAS mutation status	High concordance of KRAS mutation status between stool and tissue	Caldas 1994 [368]
DNA	Gastric cancer	Early diagnosis	The analysis of fecal DNA mutations provide diagnostic information	Youssef 2017 [369]
DNA	Melanoma, NSCL	Analysis of microbiome as predictor of immunotherapy response and toxicity	Specific microbiome compositions are predictive of immunotherapy response and toxicity	Allen-Vercoe 2020; Xu 2020; Davar 2021; Baruch 2021; Sivan 2015; Vétizou 2015 [251, 370–374]
miRNA	CRC	Early diagnosis	The expression of specific miRNAs showed a high sensitivity in early cancer detection	Wu 2012; Raut 2021; Liu 2016; Bastaminejad 2017; Phua 2014; Duran-Sanchon 2020; Duran-Sanchon 2021 [375–381]
lncRNA	CRC	Early diagnosis	cancer-related lncRNA panels to identify and distinguish CRC patients from healthy individuals	Gharib 2021 [382]

*LncRNA* Long-non-coding RNA, *miRNA* micro-RNA, *NSCLC* Non-Small Cell Lung Cancer, *CRC* Colorectal cancer

Stool DNA analysis proved its potential diagnostic utility also in patients with other tumour types particularly in pancreatic cancer that has a poor prognosis mainly due to delayed diagnosis. In particular, the analysis of KRAS mutations detected in stool samples of pancreatic patients showed a high concordance with those identified in the resected carcinomas [368]. In addition to CRC, the analysis of DNA mutations in stool specimens of gastric cancer patients demonstrating its potential application for early cancer detection [369]. Increasing evidence supports the role of the gut microbiota in the responsiveness and toxicities to immune- checkpoint inhibitors suggesting the utility of stool DNA beyond the detection of tumour DNA [251, 370–374]. Indeed, microbiome composition identified through the analysis of 16S ribosomal DNA in stool samples could act as a predictive biomarker to select patients who might benefit from immunotherapy.

Different studies have explored individual miRNAs, miRNA panels, or a combination of fecal miRNAs with fecal hemoglobin for CRC early detection [375–381]. The analysis of diagnostic performance indicators reported AUCs, sensitivities, and specificities ranging from 0.64 to 0.97, 15% to 97%, and 38% to 100%, respectively [383]. Fecal miRNAs have several advantages such as high stability and reproducibility that make them promising biomarker for CRC screening.

Although few studies have investigated the role of stool lncRNAs as potential diagnostic biomarker, some evidence reported a potential utility of cancer-related lncRNA panels to identify and distinguish CRC patients from healthy individuals [382].

In summary, the physical proximity to CRC may facilitate the detection of tumor DNA providing an optimal diagnostic tool. In this regard, stool DNA is already used for CRC screening and provides information on the genomic profiles of other tumour types such as pancreatic and gastric cancer. The major limitation is the low ctDNA component (around 0.01% of the total DNA content of stool) due to the high presence of microbial DNA [384]. Patient aversion to providing fecal samples represents another limitation which might hinder the adoption of stool liquid biopsy [385, 386].

## Conclusions

Cancer research has reached very important and advanced achievements in the last decades, by extending patient’s life and improving the quality of life for a major part of these pathologies, especially due to the development of targeted therapy, but still much more must be done.

Liquid biopsy has already revolutionized clinical practice in oncology, but it still has great hidden potential to participate in this struggle, which must be expressed by providing evidence-based guidelines for the procedure and improving the technology of this technique to maintain the integrity of the sample, by extending the cohorts of patients in its studies and the knowledge of the implications of its new biomarkers.

Several new studies and ctDNA-based trials have emerged in the last decade to investigate and expand the application of liquid biopsy in cancer management, and the promising results obtained until now indicate that it could have more important implications in different aspects of clinical practice in oncology, from diagnosis to the selection of targeted therapy and the monitoring of its effect, passing by the stratification of patients based on cancer risk and the detection of MRD.

Thus, further studies should focus also on confirming the clinical applicability of blood-based molecular profiling for CGP to improve patient outcomes.

## Data Availability

All the data generated is included within the manuscript and its supplementary files.
